# BRAF, TERT and HLA-G Status in the Papillary Thyroid Carcinoma: A Clinicopathological Association Study

**DOI:** 10.3390/ijms241512459

**Published:** 2023-08-05

**Authors:** Bruna C. Bertol, Juliana D. Massaro, Guilherme Debortoli, André L. P. Santos, Jéssica N. G. de Araújo, Tatiana M. V. Giorgenon, Matheus Costa e Silva, Nathalie L. de Figueiredo-Feitosa, Cristhianna V. A. Collares, Luiz Carlos C. de Freitas, Edson G. Soares, Luciano Neder, Vivian N. Silbiger, Rodrigo T. Calado, Léa M. Z. Maciel, Eduardo A. Donadi

**Affiliations:** 1 Postgraduate Program of Basic and Applied Immunology, Ribeirão Preto Medical School, University of São Paulo, Ribeirão Preto 14049-900, Brazil; 2 Princess Margaret Cancer Centre, University Health Network, Toronto, ON M5G 2M9, Canada; 3 Division of Clinical Immunology, Department of Medicine, Ribeirão Preto Medical School, University of São Paulo, Ribeirão Preto 14049-900, Brazil; jmassaro@alumni.usp.br (J.D.M.); matheus.costa.silva@alumni.usp.br (M.C.e.S.); kika_collares@yahoo.com (C.V.A.C.); 4 Department of Anthropology, University of Toronto, Mississauga, ON L5L 1C6, Canada; guilherme.debortoli@mail.utoronto.ca; 5 Department of Medical Imaging, Hematology, and Clinical Oncology, Ribeirão Preto Medical School, University of São Paulo, Ribeirão Preto 14049-900, Brazil; andreluizps04@gmail.com (A.L.P.S.); rtcalado@fmrp.usp.br (R.T.C.); 6 Department of Clinical Analysis and Toxicology, Federal University of Rio Grande do Norte, Natal 59012-570, Brazil; jessnaysub@hotmail.com (J.N.G.d.A.); viviansilbiger@hotmail.com (V.N.S.); 7 Division of Endocrinology and Metabolism, Department of Medicine, Ribeirão Preto Medical School, University of São Paulo, Ribeirão Preto 14049-900, Brazil; tatiana.giorgenon@usp.br (T.M.V.G.); nathaliefigueiredo@yahoo.com.br (N.L.d.F.-F.); leamaciel@hcrp.usp.br (L.M.Z.M.); 8 Department of Ophthalmology, Otorhinolaryngology and Head and Neck Surgery, Ribeirão Preto Medical School, University of São Paulo, Ribeirão Preto 14049-900, Brazil; lcconti@fmrp.usp.br; 9 Department of Pathology, Ribeirão Preto Medical School, University of São Paulo, Ribeirão Preto 14049-900, Brazil; egsoares@fmrp.usp.br (E.G.S.); neder@fmrp.usp.br (L.N.)

**Keywords:** thyroid cancer, microRNAs, polymorphisms, biomarkers

## Abstract

As BRAF, TERT, HLA-G, and microRNAs have been individually associated with papillary thyroid carcinoma (PTC), we aimed to evaluate the individual and collaborative role of these markers in PTC in the same patient cohort. HLA-G and BRAF tumor expression was evaluated by immunohistochemistry. Using molecular methods, BRAF^V600E^ and *TERT* promoter mutations were evaluated in thyroid fine needle aspirates. MicroRNA tumor profiling was investigated using massively parallel sequencing. We observed strong HLA-G (67.96%) while BRAF (62.43%) staining was observed in PTC specimens. BRAF overexpression was associated with poor response to therapy. The BRAF^V600E^ (52.9%) and *TERT*^C228T^ (13%) mutations were associated with extrathyroidal extension, advanced-age, and advanced-stage cancer. The *TERT* rs2853669 CC+TC genotypes (38%) were overrepresented in metastatic tumors. Nine modulated microRNAs targeting the *BRAF, TERT,* and/or *HLA-G* genes were observed in PTC and involved with cancer-related signaling pathways. The markers were individually associated with PTC features, emphasizing the synergistic effect of BRAF^V600E^ and *TERT*^C228T^; however, their collaborative role on PTC outcome was not fully demonstrated. The differentially expressed miRNAs targeting the *BRAF* and/or *HLA-G* genes may explain their increased expression in the tumor milieu.

## 1. Introduction

The incidence of thyroid cancer (TC) has increased worldwide during the past three decades [1]. Follicular cell-derived thyroid carcinomas are classified into the following: (i) differentiated thyroid carcinomas (DTC), such as papillary thyroid carcinoma (PTC) and follicular thyroid carcinoma (FTC); (ii) poorly differentiated thyroid carcinoma; (iii) undifferentiated anaplastic thyroid carcinoma (ATC) [2]. Although most patients with DTC are effectively treated with surgery, radioactive iodine (RAI), and thyroid-stimulating hormone (TSH) suppressive therapy, the risk of recurrence may reach up to 30%, and 5–10% of these patients develop invasive disease, distant metastases, or both [3].

The most common genetic event involved in PTC initiation is the oncogenic *1799 T>A* transversion at the *B-type Raf kinase* (*BRAF*) gene, exchanging a valine to glutamate at residue 600 of the protein (BRAF^V600E^). BRAF^V600E^ continuously activates the RAF/MEK/ERK cascade of the mitogen-activated protein kinase (MAPK) signaling pathway, increasing cellular proliferation, along with inhibition of cell differentiation and apoptosis [4]. Since the first association of BRAF^V600E^ with PTC tumorigenesis, subsequent studies have suggested further association with poor outcomes [3]. BRAF^V600E^ may reduce the expression of immune/inflammatory response genes in PTC, suggesting that immune escape mechanisms may further contribute to its pathogenesis and aggressiveness [5]. However, only scarce information on the BRAF wild type (BRAF^WT^) protein expression in PTC is available.

The telomere TTAGGG repeats are specialized structures at the end of eukaryotic chromosomes that protect DNA from degradation, end-to-end fusion, or atypical recombination [6,7]. The telomerase complex replicates telomeric DNA and helps to maintain genome integrity and replication capability in embryonic and proliferating progenitor cells; however, it is repressed in most somatic cells. The complex consists of a reverse catalytic subunit designated telomerase reverse transcriptase (TERT), which is a critical determinant in regulating the telomerase expression [8]. Two hotspot point variants at the *TERT* promoter region, located at −124 (C>T) and −146 (C>T) base pairs (bp) upstream of the translation start site, also referred to as C228T and C250T, activate *TERT* promoter activity and gene transcription in cancer [8,9], and are identified in TC with aggressive behavior, mainly when coexisting with BRAF^V600E^ [10].

Considerable attention has been devoted to the study of the inhibitory checkpoint human leukocyte antigen (HLA)-G in human cancers [11]. The HLA-G molecule can interact with leukocyte receptors (e.g., ILT-2 [LILRB1/CD85j], ILT-4 [LILRB2/CD85d], and KIR2DL4 [CD158d]), mostly on CD8^+^ T and natural killer (NK) cells, inducing inhibitory cytotoxic responses, facilitating tumor cell proliferation and spread [12]. HLA-G can be found in at least four different membrane-bound (HLA-G1 to HLA-G4) and three soluble (HLA-G5 to HLA-G7) isoforms [13]. HLA-G is not observed in healthy thyroid specimens, but may be aberrantly expressed in DTC [14], usually with clinical and pathological implications [15,16].

MicroRNAs (miRNAs) are small non-coding, highly stable RNAs that post-transcriptionally regulate gene expression. The miRNA expression pattern may be dysregulated in pathological conditions, particularly in cancer [17]. Since miRNAs are involved in the regulation of several cellular processes, such as cell cycle regulation, apoptosis, and regulation of the immune system, these molecules may be used as biomarkers and have been widely studied in cancers, including in the context of PTC development and progression [18].

Identifying unfavorable PTC cases and identifying unique or clusters of biomarkers associated with poor outcomes are of relevance in the context of tumor morbidity and prognosis. Considering that most reports have focused on the individual role of BRAF [4], TERT [19], HLA-G [14], and miRNAs [18] in PTC, in the present study, we aimed to investigate the individual and collaborative role of BRAF (tumor expression and tumor BRAF^V600E^ status), TERT (tumor *TERT* status), and HLA-G (tumor and plasma expression), as well as miRNAs (tumor expression) targeting the *HLA-G*, *BRAF* and/or *TERT* genes in the context of PTC malignancy.

Herein, we demonstrated that PTC specimens frequently expressed both HLA-G and BRAF, which were individually associated with the female sex and/or response to therapy. In this context, the differential miRNA pattern observed in tumoral areas compared to non-tumoral areas may explain the strong expression of these targets in the tumor milieu. Moreover, the BRAF^V600E^ and *TERT*^C228T^ mutations were synergistically associated with poor pathological features of PTC, including extrathyroidal invasion, more advanced cancer stage, and advanced-age at diagnosis.

## 2. Results

### 2.1. BRAF and HLA-G Expression in the Tumor

**Figure 1:**
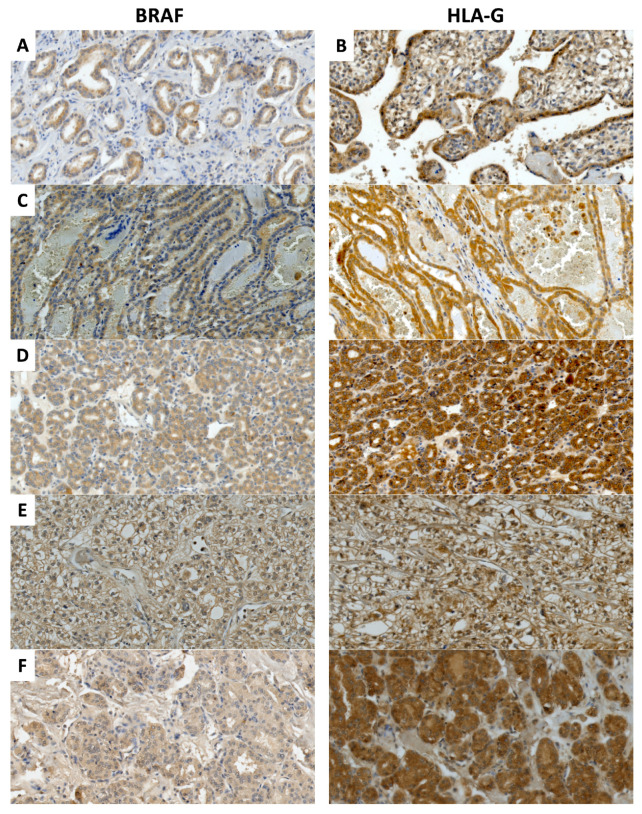
Immunohistochemical staining for BRAF (**left**) and HLA-G (**right**). Representative photomicrographs of a human prostate tumor section as a positive control for BRAF staining (**A**) and a human cytotrophoblast section from the first trimester of pregnancy as a positive control for HLA-G staining (**B**), followed by different histological subtypes of papillary thyroid carcinoma (PTC), including classic (**C**), follicular (**D**), clear cells (**E**), and oncocytic (**F**). Stained cells are shown in brown. The sections were counterstained with hematoxylin. The photomicrographs represent a 200× magnification.

HLA-G and BRAF staining was primarily observed in the cytoplasm of the PTC cells, regardless of the histological subtype (Figure 1), while most of the specimens were positive for both BRAF (88.95%) and HLA-G (97.27%). Considering the magnitude of expression, 67.96% strongly expressed HLA-G, and 62.43% strongly expressed BRAF. The HLA-G expression exhibited a median positive correlation (r = 0.428, *p* < 0.0001) with the BRAF expression (Figure S1). Female patients presented more robust expression of BRAF and HLA-G when compared to male patients, and BRAF overexpression was more frequently observed among PTC patients with poor response to conventional therapy (Table 1).

### 2.2. BRAF^V600E^ and TERT Mutations

The BRAF^V600E^ and *TERT*^C228T^ somatic mutations were found in 52.9% and 13% of the cases, respectively. When the entire cohort was stratified according to the BRAF status (BRAF^WT^ versus BRAF^V600E^), we observed that samples presenting BRAF^V600E^ also overexpressed BRAF in the tumor microenvironment—odds ratio (OR) = 4.3; 95% confidence intervals (95% CI) = 1.301–11.61. However, no significant association was observed either with PTC clinicopathological features or with HLA-G expression (Table 2). The *TERT*^C228T^ was significantly and independently associated with high-risk clinicopathological features, including extrathyroidal invasion (OR = 4.7; 95% CI = 1.262–15.54), more advanced-stage cancer at diagnosis (OR = 37; 95% CI = 6.598–177), and patients with advanced-age at tumor diagnosis (OR = 11; 95% CI = 2.431–53.17). The presence of the *TERT*^C228T^ was not associated with the magnitude of BRAF or HLA-G tissue expression (Table 2). Finally, the *TERT*^C250T^ and *TERT*^A161C^ mutations were not detected in this series.

Compared to samples without both BRAF^V600E^ and *TERT*^C228T^ mutations, (i) the presence of BRAF^V600E^ alone continued to be associated with strong BRAF expression in the tumor (OR = 3.93; 95% CI = 1.175–11.95), and (ii) the coexistence of BRAF^V600E^ and *TERT*^C228T^ mutations increased the strength of association for all the above high-risk clinicopathological features previously observed for *TERT*^C228T^, i.e., presence of extrathyroidal invasion (OR = 6.6; 95% CI = 1.302–26.12), more advanced-stage cancer at diagnosis (OR = 59.4; 95% CI = 7.232–661.3), and advanced-age at diagnosis (OR = 25.2; 95% CI = 3.693–283.7) (Table 2). However, we highlight that such associations exhibited a very broad CI. Only one sample exhibited the *TERT*^C228T^ mutation in the absence of BRAF^V600E^, impairing further assessment regarding the independent *TERT*^C228T^ implication on PTC outcome.

### 2.3. TERT Single-Nucleotide Polymorphism (SNP)

The rs2853669 *TERT* SNP was in Hardy–Weinberg equilibrium (HWE) and detected in 38% of the cases. The mutant C allele and the carriers of genotypes with at least one mutant C allele (CC+CT) were over-represented in younger patients (OR = 2.65; 95% CI = 1.087–6.925, and OR = 3.714; 95% CI = 1.311–10.53, respectively), and among those with metastasis at diagnosis (OR = 2.644; 95% CI = 1.206–5.767 and OR = 2.593; 95% CI = 1.02–6.97, respectively) (Table 3). Among the 11 cases harboring the *TERT*^C228T^ somatic mutation, only one carried the variant C of the SNP. Thus, the distribution of carriers of the variant C allele according to *TERT*^C228T^ mutational status was not assessed.

### 2.4. Soluble HLA-G

The plasma soluble HLA-G (sHLA-G) levels in PTC patients were not significantly different from controls, as previously published by our group [20]. In this study, we observed detectable sHLA-G levels in approximately half of the samples (47.1%), contrasting with the high HLA-G detection frequency (97.27%) in the tumor microenvironment. Additionally, we did not observe significant associations between plasma sHLA-G levels with (i) the magnitude of HLA-G or BRAF tissue expression, (ii) the presence or not of BRAF^V600E^, and (iii) the presence or not of *TERT*^C228T^ mutation and the *TERT* SNP (Figures S1 and S2).

### 2.5. MiRNAs Targeting BRAF, TERT, and HLA-G

A total of 39 differential expressed miRNAs were observed after comparing the PTC micro-dissected area versus the non-tumoral adjacent area. Among them, nine targeted the 3′ untranslated region (3′UTR) of the *BRAF, TERT,* and/or *HLA-G* genes, of which (i) five targeted only the *BRAF* gene (three downregulated hsa-miR-486-5p, hsa-miR-9-5p, and hsa-miR-708-3p; two upregulated hsa-miR-222-3p and hsa-miR-31-5p), (ii) two targeted only the *TERT* gene (downregulated hsa-miR-874-3p and hsa-miR-138-5p), (iii) one targeted both *BRAF* and *TERT* genes (upregulated hsa-miR-146b-3p), and (iv) only one targeted the *HLA-G* gene (downregulated hsa-miR-138-1-3p) (Table 4).

**Figure 2:**
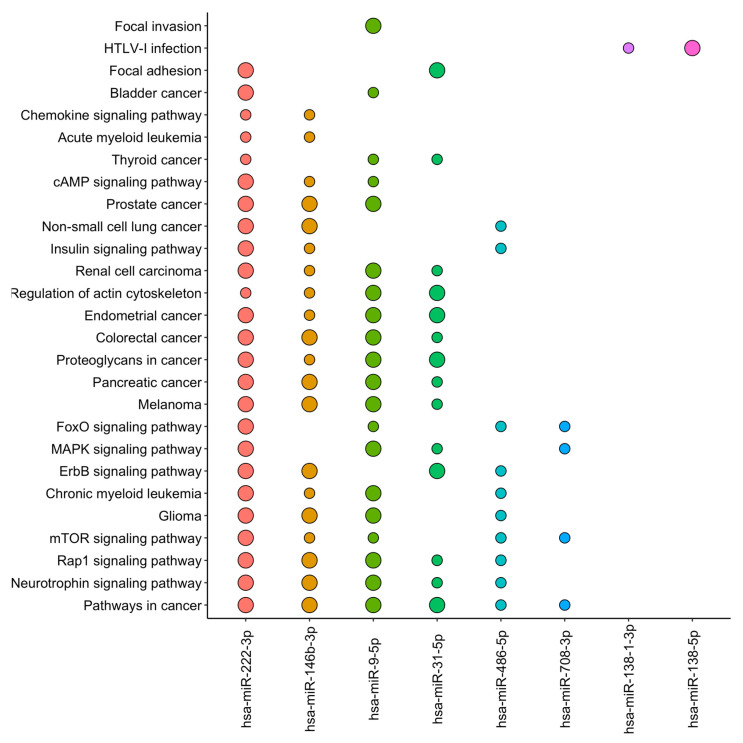
Most significant enriched pathways related to the differentially expressed miRNAs in papillary thyroid carcinoma (PTC) tumor area compared with non-tumoral adjacent area, according to the KEGG database. The enriched pathways are ordered by the number of occurrences among the miRNAs, while the miRNAs are ordered by the number of associated pathways. The larger dots represent more significant pathways considering both *p*-value ≤ 0.05 and false discovery rate (FDR) ≤ 0.05, whereas the smaller dots represent significant pathways considering only *p*-value ≤ 0.05. The hsa-miR-874-3p miRNA is absent since no significant enriched functional pathway involving the *TERT* gene target was found.

Considering only cancer-related pathways, the nine differentially expressed miRNAs participated in 27 pathways (Figure 2 and Table S1). The “pathway in cancer” was shared among six miRNAs (hsa-miR-486-5p, hsa-miR-9-5p, hsa-miR-708-3p, hsa-miR-222-3p, hsa-miR-146b-3p, and hsa-miR-31-5p). Additionally, hsa-miR-9-5p, hsa-miR-708-3p, and hsa-miR-222-3p participated in the pathway related to thyroid cancer, and in other types of human cancers (e.g., glioma, myeloid leukemia, melanoma, pancreatic cancer, etc.). The hsa-miR-222-3p was associated with a higher number of pathways, followed by hsa-miR-146b-3p and hsa-miR-9-5p.

## 3. Discussion

The search for markers associated with TC aggressiveness is a major goal in disease staging, treatment, and prognosis. We studied a group of potential markers associated with PTC pathogenesis and outcome, evaluating the relationship among them. Although the well-known BRAF^V600E^ mutation was observed in 52.9% of the cases of this series, the strongest argument against using this mutation as a single, independent prognostic and predictive factor in PTC outcome is its general high prevalence [21]. Due to its limited role for guiding patient management, the ATA guidelines [22] recommend the application of the BRAF^V600E^ status in the context of other molecular prognostic markers (such as *TERT*) to refine risk stratification and therapeutic strategy for PTC patients. In addition, BRAF protein expression may be independent of the BRAF^V600E^ mutation in TC [23]. In this study, we evaluated as follows: (i) not only the BRAF^V600E^ mutation status, but also BRAF protein expression that has been poorly explored in PTC; (ii) the *TERT* promoter mutations that are widely associated with an aberrant upregulation of TERT expression and immortalization of cancer cells [24]; (iii) the immune checkpoint HLA-G molecule expression that has been associated with poor prognosis in several human cancers [12]; (iv) the modulated miRNA profile targeting these markers in PTC areas compared with non-tumoral areas.

Considering that the antibody used for BRAF labeling does not distinguish between the BRAF^WT^ from BRAF^V600E^, we performed humoral (immunostaining in PTC specimens) and molecular (DNA extracted from fine needle aspiration [FNA] biopsy of PTC) studies to evaluate the relationship between non-mutated and mutated proteins. We observed a high frequency of BRAF expression in PTC specimens, whose magnitude of expression was significantly stronger among: (i) mutant BRAF^V600E^ positive cases, (ii) female patients—the most affected in terms of PTC incidence and mortality in worldwide populations [25], and (iii) patients exhibiting poor response to conventional therapy. Indeed, the response to therapy assessment allows a better prediction of the risk of recurrence, which is substantially higher among patients with incomplete responses [26]. Thyroidal BRAF protein overexpression in PTC has been associated with BRAF^V600E^ mutation and with extrathyroidal extension in PTC [27], as well as with lymph node metastasis [28]. In addition, high *BRAF* mRNA expression has been associated with PTC aggressiveness in both BRAF^V600E^ and BRAF^WT^ groups [29]. Taken together, these results corroborate the idea of an unfavorable role of BRAF protein overexpression, regardless of BRAF^V600E^ mutational status, indicating that the BRAF tumor expression may be helpful for PTC prognostic risk stratification.

Among the *TERT* promoter mutations evaluated in this series, the *TERT*^C228T^ was observed in only 13% of the patients, in whom the vast majority also carried the BRAF^V600E^ mutation. The *TERT*^C228T^ somatic mutation, particularly its coexistence with the BRAF^V600E^ mutation, stood out in our study as an unfavorable marker due to its association with extrathyroidal extension, more advanced-age (≥55 years) and more advanced-stage cancer (II + III + IV), similar to a previous study [30]. PTC patient age is an important predictor of outcome since tumor recurrence and death rates are significantly higher in older patients [31]. The tumor node metastasis (TNM) staging system for postoperative TC staging predicts the risk of mortality by combining age at diagnosis, size of the primary tumor, and extrathyroidal spread of the tumor; and provides prognostic information to set therapeutic strategies, enabling risk-stratified description of patients [22,32]. These results suggest that the lack of consistency of BRAF^V600E^ as an independent prognostic factor in PTC is likely due to the absence of simultaneous *TERT*^C228T^ mutation, reported to be less common in PTC. Together with previous studies [30,33], our findings suggest that BRAF^V600E^ and *TERT*^C228T^ mutations cooperatively identify the most aggressive PTC cases.

The *TERT* rs2853669 SNP has been associated with the risk and prognosis for various human tumors, although some results remain inconclusive [34]. Little attention has been devoted to its role in PTC. A Japanese cohort evaluating 58 PTC patients revealed that the SNP (all heterozygous genotype) was found in 58.6% of the cases and was associated with large tumors (>2 cm) [35]. In our study, 38% of the PTC cases exhibited the mutated allele at single or double doses (CC+TC genotypes), mainly among younger patients and in those with metastasis at diagnosis. The lymph node metastases (LNM) frequently affect young PTC patients and are associated with a high rate of local recurrence, although the mortality rates are low [36]. In addition, this SNP is associated with the prognostic value of *TERT* somatic mutations across a variety of tumor types [24]; however, larger sample sizes would be necessary to evaluate such association in PTC.

Besides the direct involvement of BRAF^V600E^ in thyroid tumor initiation and dedifferentiation, previous studies have reported an association of PTC with an immunosuppressive signature at mRNA level, as evidenced by the induction of the *CTLA-4*, *PD-L1*, and *HLA-G* genes [37]. At protein level, PTC exhibiting the BRAF^V600E^ mutation is associated with lower CD8^+^ effector to FoxP3^+^ regulatory T cells, and greater expression of PD-1 and HLA-G compared with BRAF^WT^ tumors [38]. The *TERT* mutations have also been correlated with lymphocyte infiltration, macrophage regulation, interferon (IFN)-γ, and transforming growth factor (TGF)-β response [39]. Based on these findings, we associated each of the studied mutations (individually and in combination) with tumor expression of HLA-G; however, we did not observe such associations. On the other hand, we observed a high frequency of specimens exhibiting a strong expression of HLA-G in PTC specimens, mainly among female patients. Noteworthy, sexual hormones (particularly progesterone) stimulate HLA-G expression [40].

Although normal and hyperplastic thyroid tissues may present focal and faint BRAF expression, a more diffuse expression is observed in DTC [23,28]. In parallel, HLA-G is not expressed by normal thyroid tissue; however, previous [14] and the present study have reported high frequency of HLA-G expression in the cytoplasm of PTC cells. On the other hand, the plasma sHLA-G is frequently undetectable/low in PTC patients [20,41], and its levels were not influenced by any of the assessed markers in the tumor microenvironment (BRAF or HLA-G tumor expression, BRAF^V600E^, and *TERT* mutations), as reported in this series. Therefore, the results regarding HLA-G indicate that local microenvironmental rather than peripheral mediators contribute to its increased expression in PTC specimens. Among the local mediators, miRNAs may post-transcriptionally control gene expression and serve as disease markers [17], and thus, miRNAs may be involved in the regulation of *BRAF, HLA*-*G,* and *TERT* expression in PTC.

To evaluate the role of miRNAs in PTC, we used the following strategies: (i) we separated by microdissection tumoral and non-tumoral thyroid fragments; (ii) we performed a miRNA DEA comparing both situations; (iii) after obtaining the differentially expressed miRNAs, we selected those that targeted the 3′UTR of the *BRAF*, *HLA-G*, and *TERT* genes; iv) we validated these miRNAs using an in-silico procedure. Although a formal validation by RT-qPCR was not performed, the MPS parameter was employed as a validation method, since it aims to classify the importance degree of the variables in the ability to sample differentiation.

Among the miRNAs that targeted the *BRAF* gene, the two downregulated ones (miRNA-9-5p and hsa-miR-486-5p) have been previously associated with PTC. The downregulation of miRNA-9-5p promotes proliferation of PTC cells [42], while the downregulation of hsa-miR-486-5p is associated with advanced cancer stage, LNM, recurrence, and worse overall survival [43]. The downregulation of hsa-miR-708-3p has been observed in several cancers [44,45], but its association with PTC is reported for the first time in this series. Among the induced miRNAs, the miR-31 (hsa-miR-31-5p) is upregulated in aggressive PTC [46]. The miR-222 (i.e., hsa-miR-222-3p) is commonly associated with cancer recurrence and poor outcome in PTC patients [18]. The miR-146b-3p is abundantly upregulated in PTC and has been correlated with reduced iodide uptake, which may impair the RAI treatment [47]. Although hsa-miR-146b-3p targeted both *BRAF* and *TERT* genes, only *BRAF*-related pathways were found to be statistically significant on the functional analyses.

Regarding miRNAs that targeted the *TERT* gene, the hsa-miR-874-3p has not been described in PTC, although its suppressed expression has been associated with several human solid tumor prognosis, such as osteosarcoma and gastric carcinoma [48,49]. Of note, no significant enriched functional pathway involving the *TERT* target was found for the hsa-miR-874-3p. The miR-138 (i.e., miR-138-5p) is reported to be severely decreased in TC, contributing to the overexpression of TERT, tumor stage, and an invasive phenotype [50].

Belonging to the miR-138 family, the miR-138-1-3p targets the immune checkpoint *HLA-G* gene, and it is downregulated in PTC. This downregulation is associated with tumorigenesis and LNM formation, presenting a prognostic value [51,52]. The mechanisms involved in *HLA-G* gene expression modulation are complex and have not been completely elucidated. Polymorphic sites at the *HLA-G* 5′ upstream regulatory region (5′URR) and 3′UTR may differentially target transcriptional and post-transcriptional factors associated with gene regulation. Noteworthy, sexual hormones and hypoxic factor 1, important factors associated with tumors, induce HLA-G expression [40]. Although the miR-138-1-3p has not been functionally evaluated in terms of *HLA-G* regulation, several miRNAs have the potential to target the *HLA-G* 3′UTR at polymorphic and non-polymorphic sites [40].

Considering that (i) the three upregulated miRNAs targeting *BRAF* were associated with BRAF expression/mutation in previous studies [53,54,55], (ii) the downregulation of four miRNAs in PTC tissue (three targeting *BRAF* and one targeting *HLA-G*) conducts to a harmful role in thyroid tumorigenesis, and (iii) the genes targeted by downregulated miRNAs have higher expression and increased activity on miRNA-related pathways, these findings provide a more comprehensive understanding of the complex regulation of the *BRAF* and *HLA-G* genes in PTC.

## 4. Materials and Methods

### 4.1. Studied Population

A total of 186 PTC patients (age at diagnosis 48.15 ± 15.53 years old; 150 females = 80.6%), 35 from the “Hospital Liga Norte Riograndense Contra o Câncer”, Natal-RN, Brazil, and 151 from “Hospital das Clínicas de Ribeirão Preto”, Ribeirão Preto-SP, Brazil—followed-up during a median period of 74 months (interquartile range, 45.5 to 124 months), were recruited. All patients were submitted to thyroidectomy due to or suspicion of PTC following the FNA malignant cytology of the thyroid nodule. Disease evolution during follow-up was monitored by clinical examination, ultrasound imaging, and biochemical measurement of serum thyroglobulin (Tg) and anti-Tg antibody (TgAb) levels.

The cytological and histopathological analyses were performed by experienced pathologists, as well as the TNM staging classification system according to the 8th edition of the American Joint Committee on Cancer (AJCC)/Union for International Cancer Control (UICC), ranging from stages I to IV to predict the risk of mortality for DTC. The lower the TNM stage, the lesser is the probability of cancer spread, and the lower the risk for mortality [32].

Clinical, clinicopathological data of all patients (i.e., age at diagnosis, tumor size, histological subtype, tumor invasion, metastasis at diagnosis, multicentricity, extrathyroidal extension, Hashimoto’s thyroiditis, and TNM staging), as well as treatment details (i.e., surgery approach and RAI therapy), are summarized in Table 5.

We also evaluated the clinical status of each patient at the last follow-up in terms of the individual response to initial conventional therapy, following the criteria proposed by Momesso and Tuttle, 2014 [56]; adopted by the 2015 American Thyroid Association (ATA) guidelines [22], taking into account patients treated with thyroid lobectomy or total thyroidectomy, accompanied or not by RAI ablation. The categories to evaluate response to therapy were as follows: (i) excellent response (no clinical, biochemical, or structural/imaging evidence of disease); (ii) indeterminate response (nonspecific biochemical or structural findings that require continuous observation); (iii) incomplete biochemical response (abnormal Tg or rising TgAb levels in the absence of localizable disease); (iv) incomplete structural response (persistent or newly identified by loco-regional or distant metastases, exhibiting or not abnormal Tg or TgAb). This assessment is a risk stratification system to predict the recurrence/persistence of the disease.

All subjects gave written informed consent to participate in the study, and the protocol was approved by the local Institutional Ethics Committees (process #18022/2014 and #029/029/2015).

### 4.2. Samples

Several PTC samples were collected depending on the specific use. We assessed (i) 183 PTC specimens to evaluate the HLA-G and BRAF expression, (ii) 85 plasma samples to evaluate sHLA-G expression levels, (iii) 85 DNA from FNA biopsy for BRAF^V600E^ and *TERT* promoter mutations, and (iv) eight fresh tumor fragments (all female aged 48.30 ± 15.57 years at diagnosis) to evaluate the miRNA profiles. At the pre-surgery time and following the cytological examination, the thyroid nodule aspirates were washed with phosphate buffer, and stored at −80 °C for DNA extraction. Patient blood samples were drawn on the day before surgery to obtain pre-thyroidectomy plasma, stored at −80 °C until use. During thyroidectomy, a small tumor fragment was collected, micro-dissected to separate tumoral from non-tumoral areas, placed into a microtube with TRI-Reagent^®^ (Sigma, Saint Louis, MO, USA) for immediate RNA extraction, according to the manufacturer’s protocol, and stored at −80 °C. The remaining TC specimens were used for PTC diagnosis confirmation and immunohistochemical analyses.

### 4.3. Expression of HLA-G and BRAF in the Tumor

Five µm-thick sections of paraffin-embedded TC were placed on polylysine-pretreated slides for immunohistochemical assays, using the following primary monoclonal antibody (mAb): (i) anti-HLA-G MEM-G/2 (EXBIO, Vestec, Czech Republic), which recognizes all soluble and membrane-bound HLA-G molecules, diluted at 1:100, and (ii) the anti-BRAF EP152Y (ab33899, ABCAM, Cambridge, UK), which recognizes a peptide within the human BRAF 50-150 residues, diluted at 1:250. The experiments were carried out using a biotin-free immunoenzymatic antigen detection system (REVEAL Polyvalent HRP-DAB, Spring Bioscience, Pleasanton, CA, USA), according to the manufacturer’s instructions, and slides were subsequently counterstained with hematoxylin.

As a positive control for HLA-G and BRAF expression, histological sections of human cytotrophoblast from the first trimester of pregnancy [57] and human prostate tumor [58] were used, respectively (Figure 1). Negative controls were performed omitting the primary antibody. The immunohistochemical expression was observed as brown staining. Two experienced pathologists assessed HLA-G and BRAF expression in a semiquantitative way based on the percentage (%) of stained tumor cells.

### 4.4. Expression of Soluble HLA-G

The sHLA-G plasma levels were evaluated in pre-thyroidectomy plasma, and the results together with several cytokines were previously published [20]. The sHLA-G levels were evaluated by a specific sandwich ELISA using MEM-G/9 mouse anti-human HLA-G (1:100, EXBIO) as capture mAb, and rabbit anti-human β2-microglobulin as detection mAb (1:10.000, DAKO, Santa Clara, CA, USA).

### 4.5. Identification of BRAF^V600E^ Mutation

DNA was isolated from the FNA biopsy of PTC using the QIAamp DNA Mini Kit (QIAGEN, Hilden, Germany), according to the manufacturer’s instructions. Polymerase chain reaction (PCR) was used to amplify the *BRAF* exon 15 [30]. Following amplification, the products were digested by the restriction endonuclease Xba I (Life Technologies, Carlsbad, CA, USA). BRAF^WT^ was identified by the presence of only one major fragment of 199 bp, whereas the mutant BRAF^V600E^ yielded an additional fragment of 166 bp.

### 4.6. Identification of TERT Promoter Mutations

DNA obtained from the FNA biopsy of PTC was also used to evaluate the *TERT* promoter variability, including the −245 bp T>C (rs2853669) single-nucleotide polymorphism (SNP), and the somatic mutations designated as −146 bp C>T (C250T), −124 bp C>T (C228T), and −57 bp C>T (A161C) upstream of the first ATG [59], using PCR-amplified DNA, followed by Sanger sequencing. The *TERT* promoter amplification was performed using the 5′-CAGCGCTGCCTGAAACTC-3′ and 5′-GTCCTGCCCCTTCACCTT-3′ primers. The sequencing reaction was performed using the BigDye Terminator Kit (Perkin-Elmer, Foster City, CA, USA), and the fragments were run in an ABI 3500 Genetic Analyzer DNA Sequencer (Thermo Fisher Scientific, Waltham, MA, USA).

### 4.7. Statistical Analysis

The immunoreactivity of BRAF or HLA-G in the tumor was stratified into “weak” (≤50%) or “strong” (>50%) cell staining. Thus, qualitative/categorical data, including the magnitude of HLA-G/BRAF expression, the BRAF^V600E^ and *TERT* promoter mutations were analyzed using the chi-squared (χ^2^) test. The OR and 95% CI were also estimated to assess the strength of the associations.

The plasma levels of sHLA-G were assessed by both qualitative (detection) and quantitative (levels) approaches. We stratified sHLA-G levels of PTC patients into two groups (undetectable or detectable levels) to explore the relationship between qualitative variables using the chi-squared (χ^2^) test. For the quantitative approach, we used the unpaired nonparametric Mann–Whitney test to compare the median levels of sHLA-G in PTC patients stratified according to (i) the magnitude of HLA-G/BRAF tissue expression, and (ii) BRAF^V600E^ and *TERT* promoter tumor mutations. Correlations were evaluated using the non-parametric Spearman correlation coefficient (r).

Analyses were performed using GraphPad Prism Software (version 7, La Jolla, CA, USA) and for all tests, a 5% level of significance (α = 0.05) was considered for rejection of the null hypothesis. Regarding the *TERT* SNP, adherences of genotypic proportions to expectations under the Hardy–Weinberg equilibrium (HWE) were tested by the exact test of Guo and Thompson [60] using ARLEQUIN 3.5 software [61].

### 4.8. MiRNA Expression Profiling

The miRNA profiling was performed using massively parallel sequencing, as previously described [62]. Only samples presenting an RNA Integrity Number (RIN) ≥ 8.0 were used for further analyses. The small RNA libraries were produced using TruSeq Small RNA Library Preparation Kit (Illumina, San Diego, CA, USA), following the manufacturer’s instructions. The pooled libraries were quantified by quantitative reverse transcription PCR, using the KAPA Library Quantification Kit (KAPA Biosystems, Wilmington, MA, USA), and sequenced using MiSeq Reagent Kit v2 (50 cycles) on the MiSeq system (Illumina).

### 4.9. Bioinformatic Analysis for miRNA

The fastq files were submitted to a data-processing pipeline that included several steps as previously reported [62]. Differential expression analysis (DEA) was performed using the *R*Leave protocol (downloaded at: arxiv.org/abs/2012.05421 accessed on 3 August 2023; registered at: zenodo.org/record/3365736 accessed on 3 August 2023). This protocol includes the classical analysis of edgeR package followed by leave-one-out and decision tree approaches, which provide in addition to the usual parameters (*p*-value, false discovery rate [FDR] for multiple testing correction and log-fold-change [logFC]), a maximum prevalence score (MPS). The latter parameter discloses the sum of how many times a differentially expressed miRNA appeared throughout all analyses, divided by the total number of analyses, and this approach can be considered an in-silico validation test. Therefore, most significant differentially expressed miRNAs were considered when (i) *p*-values and FDR ≤ 0.05, (ii) logFC values > 1.5, and (iii) MPS ≥ 0.5.

Public databases were used to verify miRNA–mRNA interactions for target prediction as previously described [62]. Only miRNAs targeting *BRAF*, *TERT,* and/or *HLA-G* genes were selected for this study. For functional enrichment analysis, the list of genes obtained from the target predictions, including our targets of interest, was uploaded into DAVID v6.7 [63], taking into account the biological pathway (KEGG_PATHWAY) parameters exhibiting EASE threshold ≤ 0.05 and count threshold = 3. The figure of the most significant KEGG enriched pathways was constructed using the package ggplot2, implemented in the R statistical environment [64,65].

## 5. Conclusions

We reported that each of the HLA-G, BRAF, and TERT markers may be independently used to identify poor clinicopathological characteristics of PTC, including the potential synergistic unfavorable effect of BRAF^V600E^ and *TERT*^C228T^. We were not able to fully demonstrate a cooperative role of all the three markers for PTC prognosis. We identified differentially expressed miRNAs present in the PTC microenvironment that (i) target the *BRAF*, *TERT*, and/or *HLA-G* genes, (ii) are involved in important cancer associated-signaling pathways in PTC pathogenesis, and (iii) at least in part, may explain the increased expression of these genes/proteins in the tumor milieu.

## Figures and Tables

**Table 1:** Expression of BRAF and HLA-G in PTC specimens according to demographic and clinicopathological data.

Demographic and Clinicopathological Data		BRAF (*n* = 181)						HLA-G (*n* = 183)					
			≤50%		>50%				≤50%		>50%		
			*n* = 68		*n* = 113				*n* = 59		*n* = 124		
		*n*	*n*	%	*n*	%	*p*	*n*	*n*	%	*n*	%	*p*
Sex	Female	145	49	72.1	96	84.96	0.0353	147	41	69.49	106	85.48	0.011
	Male	36	19	27.9	17	15.04		36	18	30.51	18	14.52	
Age at diagnosis (years old)	<55	124	49	72.1	75	66.37	0.425	124	39	66.1	85	68.55	0.7407
	≥55	57	19	27.9	38	33.63		59	20	33.9	39	31.45	
Tumor size (cm)	<2	103	39	57.4	64	56.64	0.925	103	35	59.32	68	54.84	0.5677
	≥2	78	29	42.6	49	43.36		80	24	40.68	56	45.16	
Histological subtype	Classic	89	31	45.6	58	51.33	0.4545	90	33	55.93	57	45.97	0.2076
	Others ^#^	92	37	54.4	55	48.67		93	26	44.07	67	54.03	
Tumor invasion	Absent	129	50	73.5	79	69.91	0.6024	131	40	67.8	91	73.39	0.4332
	Present	52	18	26.5	34	30.09		52	19	32.2	33	26.61	
Extrathyroidal extension	Absent	132	52	76.5	80	70.8	0.4054	132	44	74.58	88	70.97	0.6108
	Present	49	16	23.5	33	29.2		51	15	25.42	36	29.03	
Multicentricity	Absent	114	40	58.8	74	65.49	0.3686	116	37	62.71	79	63.71	0.8958
	Present	67	28	41.2	39	34.51		67	22	37.29	45	36.29	
Metastasis at diagnosis	Absent	128	45	66.2	83	73.45	0.2975	129	38	64.41	91	73.39	0.2131
	Present	53	23	33.8	30	26.55		54	21	35.59	33	26.61	
Hashimoto’s thyroiditis	Absent	130	48	70.6	82	72.57	0.7745	132	43	72.88	89	71.77	0.8759
	Present	51	20	29.4	31	27.43		51	16	27.12	35	28.23	
TNM staging 8th	I	148	53	77.94	95	85.6	0.1896	148	45	76.3	103	84.4	0.1829
	II/III/IV	31	15	22.06	16	14.4		33	14	23.7	19	15.6	
	Unknown	2	-	-	-	-		2	-	-	-	-	
Response to therapy	Excellent	77	33	68.8	44	50	0.035	77	21	58.33	56	56	0.8086
	Others ^◊^	59	15	31.3	44	50		59	15	41.67	44	44	
	Unknown	45	-	-	-	-		47	-	-	-	-	

PTC: Papillary thyroid carcinoma. TNM: tumor node metastasis. ≤50%: Equal or less than 50% of stained thyroid tumor cells. >50%: Over 50% of stained thyroid tumor cells. The BRAF expression was not assessed in 2/183 thyroid tumor specimens due to technical issues. Comparisons were made by using chi-squared (χ^2^) test. ^#^ Follicular, oncocytic, solid, trabecular, and others. ^◊^ The sum of patients presenting indeterminate, biochemical, or structural incomplete response.

**Table 2:** BRAF and *TERT* mutations in FNA biopsy of PTC, stratified according to demographic and clinicopathological data; and according to HLA-G and BRAF tumor expression.

Demographic and Clinicopathological Data			Overall Analysis of Each Mutation												Analysis of Mutations Alone ^ϕ^ or in Coexistence								
			BRAF (*n* = 85)						*TERT* (*n* = 84)						No Mutation *n* = 38		BRAF^V600E^ only *n* = 35			BRAF^V600E^ + *TERT*^C228T^ *n* = 10			
				WT *n* = 40		V600E *n* = 45				WT *n* = 73		C228T *n* = 11											
			*n*	*n*	%	*n*	%	*p*	*n*	*n*	%	*n*	%	*p*	*n*	%	*n*	%	*p* *	*n*	%	*p* *	
	Sex	Female	72	32	80.00	40	88.89	0.2558	71	62	84.93	9	81.82	0.7901	30	78.95	32	91.43	0.1364	8	80.00	0.9419	
		Male	13	8	20.00	5	11.11		13	11	15.07	2	18.18		8	21.05	3	8.57		2	20.00		
	Age at diagnosis (Years old)	<55	55	30	75.00	25	55.56	0.0610	54	52	71.23	2	18.18	0.0006	28	73.68	24	68.57	0.6297	1	10.00	0.0002	
		≥55	30	10	25.00	20	44.44		30	21	28.77	9	81.82		10	26.32	11	31.43		9	90.00		
	Tumor size (cm)	<2	43	18	45.00	25	55.56	0.3310	43	38	52.05	5	45.45	0.6831	17	44.74	21	60.00	0.1922	4	40.00	0.7882	
		≥2	42	22	55.00	20	44.44		41	35	47.95	6	54.55		21	55.26	14	40.00		6	60.00		
	Histological subtype	Classic	32	11	27.50	21	46.67	0.0690	32	28	38.36	4	36.36	0.8991	11	28.95	17	48.57	0.0850	4	40.00	0.5023	
		Others ^#^	53	29	72.50	24	53.33		52	45	61.64	7	63.64		27	71.05	18	51.43		6	60.00		
	Tumor invasion	Absent	52	23	57.50	29	64.44	0.5120	52	47	64.38	5	45.45	0.2281	23	60.53	24	68.57	0.4733	5	50.00	0.5480	
		Present	33	17	42.50	16	35.56		32	26	35.62	6	54.55		15	39.47	11	31.43		5	50.00		
	Extrathyroidal extension	Absent	69	35	87.50	34	75.56	0.1600	68	62	84.93	6	54.55	0.0167	33	86.84	29	82.86	0.6345	5	50.00	0.0107	
		Present	16	5	12.50	11	24.44		16	11	15.07	5	45.45		5	13.16	6	17.14		5	50.00		
	Multicentricity	Absent	47	20	50.00	27	60.00	0.3550	46	39	53.42	7	63.64	0.5259	19	50.00	20	57.14	0.5411	7	70.00	0.2587	
		Present	38	20	50.00	18	40.00		38	34	46.58	4	36.36		19	50.00	15	42.86		3	30.00		
	Metastasis at diagnosis	Absent	59	27	67.50	32	71.11	0.7180	58	53	72.60	5	45.45	0.0694	26	68.42	27	77.14	0.4039	5	50.00	0.2785	
		Present	26	13	32.50	13	28.89		26	20	27.40	6	54.55		12	31.58	8	22.86		5	50.00		
	Hashimoto’s thyroiditis	Absent	58	24	60.00	34	75.56	0.1240	58	52	71.23	6	54.55	0.2644	24	63.16	28	80.00	0.1123	6	60.00	0.8544	
		Present	27	16	40.00	11	24.44		26	21	28.77	5	45.45		14	36.84	7	20.00		4	40.00		
	TNM staging 8th	I	68	35	87.50	33	73.33	0.1030	67	65	89.04	2	18.18	<0.0001	33	86.84	32	91.43	0.5309	1	10.00	<0.0001	
		II/III/IV	17	5	12.50	12	26.67		17	8	10.96	9	81.82		5	13.16	3	8.57		9	90.00		
	Response to therapy	Excellent	42	20	50.00	22	48.89	0.9190	41	36	49.32	5	45.45	0.8113	19	50.00	17	48.57	0.9029	5	60	1.0000	
		Others ^◊^	43	20	50.00	23	51.11		43	37	50.68	6	54.55		19	50.00	18	51.43		5	40		
**Protein expression**																							
	HLA-G	≤50%	19	10	25.64	9	20.93	0.6136	19	16	22.86	3	27.27	0.7480	10	27.03	6	18.18	0.3790	3	30.00	0.8521	
		>50%	63	29	74.36	34	79.07		62	54	77.14	8	72.73		27	72.97	27	81.82		7	70.00		
		Unknown	3	-	-	-	-		3	-	-	-	-		1	-	2	-		0	-		
	BRAF	≤50%	19	14	35.90	5	11.63	0.0093	18	17	24.29	1	9.09	0.2598	13	35.14	4	12.12	0.0250	1	10.00	0.1231	
		>50%	63	25	64.10	38	88.37		63	53	75.71	10	90.91		24	64.86	29	87.88		9	90.00		
		Unknown	3	-	-	-	-		3	-	-	-	-		1	-	2	-		0	-		

PTC: Papillary thyroid carcinoma. FNA: Fine needle aspiration. TNM: tumor node metastasis. WT: Wild-type. ≤50%: Equal or less than 50% of stained thyroid tumor cells. >50%: Over 50% of stained thyroid tumor cells. The *TERT* promoter mutation was not assessed in 1/85 thyroid sample due to technical issues. Comparisons were made by using chi-squared (χ^2^) test. *p*-values * compares “no mutation” to “BRAF^V600E^ only” and to “BRAF^V600E^ + *TERT*^C228T^”. ^ϕ^ There is no “*TERT*^C228T^ only” column since there was only one sample in this category, thus, *n* = 83 for the analysis of the mutations alone or in coexistence. ^#^ Follicular, oncocytic, solid, trabecular, and others. ^◊^ The sum of patients presenting indeterminate, biochemical, or structural incomplete response.

**Table 3:** *TERT* rs2853669 polymorphism in FNA biopsy of PTC stratified according to demographic and clinicopathological data (*n* = 84).

Demographic and Clinicopathological Data		Genotypes (*n* = 84)						Alleles (2*n* = 168)					
			TT *n* = 52		CC+CT *n* = 32				T 2*n* = 133		C 2*n* = 35		
		*n*	*n*	%	*n*	%	*p*	2*n*	*n*	%	*n*	%	*p*
Sex	Female	71	43	82.69	28	87.50	0.5541	142	112	84.21	30	85.71	0.8268
	Male	13	9	17.31	4	12.50		26	21	15.79	5	14.29	
Age at diagnosis (Years old)	<55	54	28	53.85	26	81.25	0.0109	108	80	60.15	28	80.00	0.0292
	≥55	30	24	46.15	6	18.75		60	53	39.85	7	20.00	
Tumor size (cm)	<2	43	26	50.00	17	53.13	0.7808	86	67	50.38	19	54.29	0.6805
	≥2	41	26	50.00	15	46.88		82	66	49.62	16	45.71	
Histological subtype	Classic	32	23	44.23	9	28.13	0.1399	64	54	40.60	10	28.57	0.1922
	Others ^#^	52	29	55.77	23	71.88		104	79	59.40	25	71.43	
Tumor invasion	Absent	52	31	59.62	21	65.63	0.5818	104	82	61.65	22	62.86	0.8963
	Present	32	21	40.38	11	34.38		64	51	38.35	13	37.14	
Extrathyroidal extension	Absent	68	41	78.85	27	84.38	0.5309	136	106	79.70	30	85.71	0.4201
	Present	16	11	21.15	5	15.63		32	27	20.30	5	14.29	
Multicentricity	Absent	46	29	55.77	17	53.13	0.8131	92	74	55.64	18	51.43	0.656
	Present	38	23	44.23	15	46.88		76	59	44.36	17	48.57	
Metastasis at diagnosis	Absent	58	40	76.92	18	56.25	0.0466	116	98	73.68	18	51.43	0.0113
	Present	26	12	23.08	14	43.75		52	35	26.32	17	48.57	
Hashimoto’s thyroiditis	Absent	58	36	69.23	22	68.75	0.9631	116	93	69.92	23	65.71	0.6316
	Present	26	16	30.77	10	31.25		52	40	30.08	12	34.29	
TNM staging 8th	I	67	39	75.00	28	87.50	0.1661	134	103	77.44	31	88.57	0.1449
	II/III/IV	17	13	25.00	4	12.50		34	30	22.56	4	11.43	
Response to therapy	Excellent	41	26	50.00	15	46.88	0.7810	82	65	48.87	17	48.57	0.9750
	Others ^◊^	43	26	50.00	17	53.13		86	68	51.13	18	51.43	

PTC: Papillary thyroid carcinoma. FNA: Fine needle aspiration. TNM: tumor node metastasis. The *TERT* promoter SNP was not assessed in 1/85 thyroid sample due to technical issues. Comparisons were made by using chi-squared (χ^2^) test. ^#^ Follicular, oncocytic, solid, trabecular, others. ^◊^ The sum of patients presenting indeterminate, biochemical, or structural incomplete response.

**Table 4:** Most representative differentially expressed miRNAs targeting *BRAF*, *TERT,* and/or *HLA-G* observed in PTC tumoral area in comparison to non-tumoral adjacent area.

miRNA	Previous ID ^#^	Accession Number ^#^	Target(s)	logFC	*p*	FDR	Regulation	MPS
hsa-miR-486-5p	hsa-miR-486	MIMAT0002177	*BRAF*	−3.20	5.50 × 10^−6^	0.0004	DOWN	1
hsa-miR-874-3p	hsa-miR-874	MIMAT0026718	*TERT*	−2.05	5.24 × 10^−5^	0.0020	DOWN	1
hsa-miR-138-1-3p	hsa-miR-138-1	MIMAT0004607	*HLA-G*	−2.31	1.52 × 10^−5^	0.0008	DOWN	1
hsa-miR-138-5p	hsa-miR-138	MIMAT0000430	*TERT*	−2.39	0.0002	0.0054	DOWN	1
hsa-miR-9-5p	hsa-miR-9	MIMAT0000441	*BRAF*	−2.95	0.0002	0.0057	DOWN	0.94
hsa-miR-708-3p	hsa-miR-708	MIMAT0004927	*BRAF*	−1.72	0.0007	0.0137	DOWN	1
hsa-miR-222-3p	hsa-miR-222	MIMAT0000279	*BRAF*	2.77	7.54 × 10^−6^	0.0004	UP	1
hsa-miR-146b-3p	-	MIMAT0004766	*BRAF/TERT*	4.38	4.61 × 10^−7^	8.12 × 10^−5^	UP	1
hsa-miR-31-5p	hsa-miR-31	MIMAT0000089	*BRAF*	4.21	4.34 × 10^−8^	1.53 × 10^−5^	UP	1

PTC: Papillary thyroid carcinoma. The most representative miRNAs are shown based on the following criteria: *p*-value ≤ 0.05, log-fold-change (logFC) ≥ 1.5, false discovery rate (FDR) ≤ 0.05, and maximum prevalence score (MPS) ≥ 0.5. Regulation: UP (induced) and DOWN (repressed). ^#^ Based on miRBase database.

**Table 5:** Clinical and histopathological features of the PTC patients included in the study (*n* = 186).

	*N*	%
Surgical strategy		
Total thyroidectomy	184	98.9
Information not available	2	1.1
Radioactive iodine (RAI) therapy		
Yes	153	82.3
No	31	16.7
Information not available	2	1.1
Age at diagnosis (years old) (mean: 48.15)		
<55	126	67.7
≥55	60	32.3
Tumor size (cm) (mean: 2.15)		
<2	106	57.0
≥2	80	43.0
Histological subtype		
Classic	92	49.5
Follicular	44	23.7
Oncocytic	17	9.1
Classical and follicular	13	7.0
Follicular and oncocytic	5	2.7
Follicular and solid	5	2.7
Solid	3	1.6
Trabecular	2	1.1
Others	5	2.5
Tumor invasion		
Absent	134	72.0
Present:	52	28.0
Angiolymphatic	17	9.1
Vascular	12	6.5
Capsular	10	5.4
Capsular and vascular	3	1.6
Angiolymphatic and capsular	3	1.6
Angiolymphatic and perineural	3	1.6
Vascular and angiolymphatic	2	1.1
Vascular and perineural	1	0.5
Perineural	1	0.5
Extrathyroidal extension ^a^		
Absent	133	71.5
Present	53	28.5
Multicentricity		
Absent	118	63.4
Present	68	36.6
Metastasis at diagnosis		
Absent	132	71.0
Present:	54	29.0
Lymph node (s)	48	25.8
Lymph node (s) and lung	4	2.2
Lymph node (s), lung and bone	1	0.5
Lung	1	0.5
Hashimoto’s thyroiditis		
Absent	134	72.0
Present	52	28.0
TNM staging 8th ^b^		
I	151	81.2
II	25	13.4
III	3	1.6
IV	5	2.7
Information not available	2	1.1
Response to therapy ^c^		
Excellent	78	41.9
Indeterminate	32	17.2
Biochemical incomplete	13	7.0
Structural incomplete	16	8.6
Information not available	47	25.3

^a^ Including minimal (immediate perithyroidal soft tissues or sternothyroid muscle typically detected only microscopically) and extensive (subcutaneous soft tissues, i.e., larynx, trachea, esophagus, or recurrent laryngeal nerve) extrathyroidal extension. ^b^ At initial diagnosis. Tumor node metastasis (TNM) system refers to the primary tumor, lymph node metastasis and distant metastasis. ^c^ Classification at the final follow-up.

## Data Availability

The data can be requested from the corresponding authors.

## Supplementary Materials

The following supporting information can be downloaded at: https://www.mdpi.com/article/10.3390/ijms241512459/s1.
