# Supplementary material for: BRAF, TERT and HLA-G Status in the Papillary Thyroid Carcinoma: A Clinicopathological Association Study
Source: Int J Mol Sci. 2023 Aug 5;24(15):12459. doi: 10.3390/ijms241512459 (PMC10419559; doi:10.3390/ijms241512459)
Supplement: Supplementary file 1 [file ijms-24-12459-s001.zip › ijms-2534345-supplementary.pdf]

SUPPLEMENTARY MATERIAL

Article

BRAF, TERT and HLA-G status in the papillary thyroid carcinoma: A clinicopathological association study

Bruna C. Bertol [1,2,*], Juliana D. Massaro [3], Guilherme Debortoli [4], André L. P. Santos [5], Jéssica N. G. de Araújo [6], Tatiana M. V. Giorgenon [7], Matheus Costa e Silva [3], Nathalie L. de Figueiredo-Feitosa [7], Cristhianna V. A. Collares [3], Luiz Carlos C. de Freitas [8], Edson G. Soares [9], Luciano Neder [9], Vivian N. Silbiger [6], Rodrigo T. Calado [5], Léa M. Z. Maciel [7] and Eduardo A. Donadi [1,3,*]

[1] Postgraduate Program of Basic and Applied Immunology, Ribeirão Preto Medical School, University of São Paulo, Ribeirão Preto 14049-900, Brazil.
[2] Princess Margaret Cancer Centre, University Health Network, Toronto, ON M5G 2M9, Canada.
[3] Division of Clinical Immunology, Department of Medicine, Ribeirão Preto Medical School, University of São Paulo, Ribeirão Preto 14049-900, Brazil.
[4] Department of Anthropology, University of Toronto, Mississauga, ON L5L 1C6, Canada.
[5] Department of Medical Imaging, Hematology, and Clinical Oncology, Ribeirão Preto Medical School, University of São Paulo, Ribeirão Preto 14049-900, Brazil.
[6] Department of Clinical Analysis and Toxicology, Federal University of Rio Grande do Norte, Natal 59012-570, Brazil.
[7] Division of Endocrinology and Metabolism, Department of Medicine, Ribeirão Preto Medical School, University of São Paulo, Ribeirão Preto 14049-900, Brazil.
[8] Department of Ophthalmology, Otorhinolaryngology and Head and Neck Surgery, Ribeirão Preto Medical School, University of São Paulo, Ribeirão Preto 14049-900, Brazil.
[9] Department of Pathology, Ribeirão Preto Medical School, University of São Paulo, Ribeirão Preto 14049-900, Brazil.
* Correspondence: bruna.bertol@uhn.ca (B.C.B.); eadonadi@fmrpusp.br (E.A.D.); Tel.: +55-16-3602-2566 (E.A.D.)

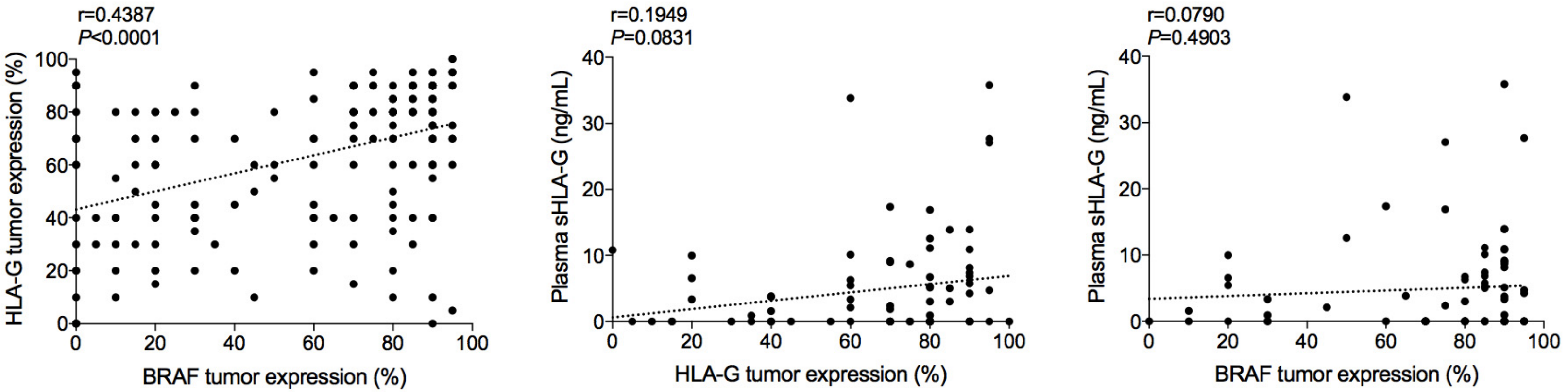

Figure S1. Correlation analysis between (i) HLA-G and BRAF tissue expression in papillary thyroid carcinoma (PTC) specimens based on the percentage (%) of stained thyroid tumor cells (left) (n = 181); (ii) soluble HLA-G (sHLA-G) plasma levels in PTC patients and HLA-G expression in PTC specimens based on the percentage (%) of stained thyroid tumor cells (middle) (n = 80) and (iii) sHLA-G plasma levels in PTC patients and BRAF expression in PTC specimens based on the percentage (%) of stained thyroid tumor cells (right) (n = 78). Spearman correlation coefficient (r) and p -values are shown.

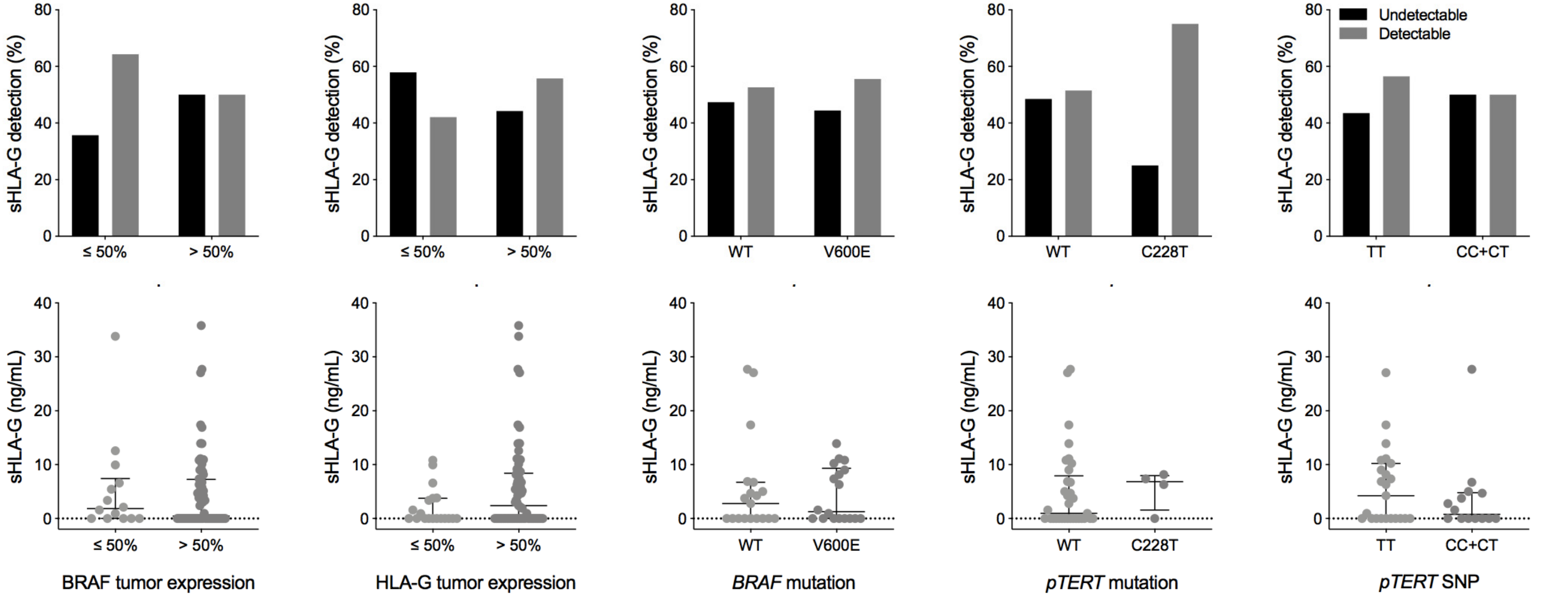

Figure S2. Plasma soluble HLA-G (sHLA-G) detection frequency (top) and levels (bottom) in papillary thyroid carcinoma (PTC) according to (i) the magnitude of HLA-G and BRAF tumor expression, and (ii) BRAF^{V600E} and *TERT* promoter mutations. Top: Comparisons were made by using Chi-Squared (χ^2) test. Bottom: Each dot represents one plasma sample. Results are shown as median with interquartile range. Comparisons were made by using unpaired Mann-Whitney test. No significant difference (p-value below 0.0500) was observed. *pTERT*: *TERT* promoter.

Table S1. Most significant enriched pathways related to the differentially expressed miRNAs in papillary thyroid carcinoma (PTC) tumor area compared with non-tumoral adjacent area, according to the KEGG database.

KEGG ID	Pathway	*P*-value	FDR	All target genes within the pathway (including the genes of interest in *bold*)
				**hsa-miR-486-5p**
hsa04150	mTOR signaling pathway	5.06e-04	0.1431	*PRKCA, PIK3CG, **BRAF**, IGF1, RPS6KB1, RICTOR, PTEN, PRKCB, DDIT4, RPS6KA6, RPS6KA3, PDPK1, EIF4E, CAB39, MLST8, PIK3R3, EIF4E2, PIK3R1*
hsa04910	Insulin signaling pathway	0.0059	0.2840	*PRKAG3, PHKB, PRKAG2, MKNK1, FOXO1, RHOQ, ELK1, RPS6KB1, PPP1R3E, PDPK1, PPP1R3B, SORBS1, PIK3R3, RAPGEF1, PIK3R1, SHC4, PIK3CG, **BRAF**, PRKAB1, PPARGC1A, G6PC, EIF4E, CRKL, PRKAR1A, ARAF, PTPN1, CRK, EIF4E2, CALM2*
hsa04068	FoxO signaling pathway	0.0075	0.2840	*PRKAG3, PRKAG2, FOXO1, FASLG, PTEN, IL10, IGF1R, PDPK1, FBXO25, PIK3R3, AGAP2, PIK3R1, PIK3CG, **BRAF**, SGK3, TGFBR1, PRKAB1, IGF1, SMAD2, C8ORF44-SGK3, SIRT1, STK4, BCL2L11, STAT3, G6PC, ARAF, MDM2, FBXO32*
hsa05214	Glioma	0.0125	0.3409	*PRKCA, PIK3CG, **BRAF**, IGF1, CDK4, PTEN, PRKCB, IGF1R, PLCG1, ARAF, MDM2, PIK3R3, CAMK2A, PIK3R1, CALM2, SHC4*
hsa04012	ErbB signaling pathway	0.0202	0.3409	*PRKCA, PIK3CG, NRG4, **BRAF**, ELK1, RPS6KB1, PRKCB, PTK2, CRKL, PLCG1, ARAF, NRG1, PIK3R3, MAP2K7, CAMK2A, MYC, CRK, PIK3R1, SHC4*
hsa04722	Neurotrophin signaling pathway	0.0228	0.3409	*PIK3CG, **BRAF**, FASLG, KIDINS220, IRAK4, CDC42, RPS6KA6, PDPK1, RPS6KA3, CRKL, PSEN1, PLCG1, MAP3K1, SORT1, RAP1A, NGFR, PIK3R3, MAP2K7, CAMK2A, RAPGEF1, CRK, CALM2, PIK3R1, SHC4*
hsa04015	Rap1 signaling pathway	0.0287	0.3799	*F2RL3, GNAI3, FGF7, FGF9, EFNA1, CSF1, EFNA3, FPR1, FGF13, IGF1R, CDC42, PFN2, RAPGEF5, PDGFC, PIK3R3, RAPGEF1, PIK3R1, MAP2K6, FGF4, PRKCA, PIK3CG, FLT1, MAGI2, **BRAF**, GRIN2A, IGF1, PRKCB, CRKL, PLCG1, RAP1A, GNAS, PARD6G, NGFR, CRK, PRKD3, CALM2, F2R*
hsa05220	Chronic myeloid leukemia	0.0305	0.3799	*PIK3CG, CTBP2, **BRAF**, TGFBR1, MECOM, CDK4, CRKL, GAB2, HDAC1, ARAF, MDM2, PIK3R3, MYC, CRK, PIK3R1, SHC4*
hsa05200	Pathways in cancer	0.0402	0.4647	*F2RL3, FGF7, FGF9, ARNT2, FASLG, FOXO1, FGF13, PTEN, CXCL12, CDC42, RARA, TPR, WNT6, MYC, FGF4, PRKCA, PIK3CG, CTBP2, **BRAF**, ROCK2, RXRB, RUNX1T1, FADD, CTNNA1, CDK4, MECOM, STK4, PRKCB, CRKL, MDM2, GNB4, GNAS, LAMC2, WNT9A, DCC, GNAI3, WNT5B, XIAP, GNG12, TPM3, RBX1, IGF1R, PTK2, PIK3R3, TRAF4, PIK3R1, APC, COL4A4, MSH3, VHL, TGFBR1, ITGA2, IGF1, SMAD2, BIRC3, STAT3, PLCG1, HDAC1, ETS1, ARAF, CRK, F2R*
hsa05223	Non-small cell lung cancer	0.0410	0.4647	*PRKCA, PIK3CG, FHIT, PDPK1, **BRAF**, PLCG1, RXRB, ARAF, PIK3R3, CDK4, STK4, PIK3R1, PRKCB*
				**hsa-miR-138-1-3p**
hsa05166	HTLV-I infection	0.0410	0.5924	*IL1R2, E2F2, ADCY2, ADCY7, CRTC1, ELK1, CTNNB1, ATF2, XBP1, PIK3R3, NFATC2, HLA-DOB, FZD9, PIK3CG, KAT2B, EGR2, CREB1, TP53, SMAD2, FZD2, FZD5, FZD4, FZD7, **HLA-G**, DVL1, POLD3, ETS1, CCND2*
				**hsa-miR-138-5p**
hsa05166	HTLV-I infection	0.0035	0.0363	*E2F2, ADCY1, E2F3, NRP1, PDGFB, STAT5B, NFKB1, AKT1, WNT1, WNT3, CDKN2B, SLC2A1, IL15RA, PRKACA, PRKACB, FOSL1, MYC, **TERT**, AKT3, AKT2, DLG1, ICAM1, RELA, POLE, HLA-DQA2, CCND1, EP300, CCND3, CCND2, LCK, WNT9B, PDGFRB, ANAPC7, WNT9A, ITGAL, IL1R1, XIAP, CREM, MRAS, PPP3R2, ATF1, TNFRSF1A, KRAS, MAP3K3, PPP3CB, HLA-DPB1, HLA-DOA, PIK3R3, NFATC2, TCF3, PIK3R1, NFATC1, DVL2, DVL3, VAC14, CREB1, SMAD4, SMAD3, SMAD2, VDAC2, CDKN1A, GSK3B, IKBKG, TP53INP1*
				**hsa-miR-9-5p**
hsa05200	Pathways in cancer	4.20e-11	9.95e-09	*GNA13, FGF18, ADCY1, FGF14, MMP9, ADCY5, ADCY6, FGF10, FOXO1, FGF12, LPAR1, MMP2, CXCL12, MMP1, TGFB2, WNT4, CDKN2A, RALB, PRKACB, WNT6, RET, **BRAF**, ROCK1, PIK3CB, RXRA, CTNNA1, CTNNA3, JUP, CCDC6, EP300, JUN, VEGFA, PDGFRB, LAMC2, LAMC1, FGFR1, GNAI1, ARNT, ITGAV, RAC1, NKX3-1, RUNX1, TRAF3, FN1, TCF7, EPAS1, TGFBR1, TGFBR2, MET, SMAD4, ITGA3, LAMA4, HDAC2, ITGA6, ETS1, FGF5, PPARD, E2F3, FGF9, SPI1, NFKB1, PTEN, CCNE2, CDC42, CCNE1, PLCB4, CXCR4, PAX8, RHOA, PLCB1, AKT3, PRKCA, AR, HSP90AA1, RUNX1T1, TP53, CDK6, ARHGEF12, RAD51, DAPK1, CCND1, GNAQ, MDM2, GNAS, DCC, EGLN3, KITLG, CDH1, SUFU, TCF7L1, TPM3, IGF1R, SOS1, BCL2, RASGRP1, SOS2, PIK3R3, WNT8A, FZD8, COL4A2, IL6, COL4A1, MSH2, CBL, IGF1, FZD3, FZD5, RALGDS, FZD6, ADCY9, GSK3B, JAK1, PTCH1*

KEGG ID	Pathway	*P*-value	FDR	All target genes within the pathway (including the genes of interest in *bold*)
hsa04510	Focal invasion	2.26e-08	2.68e-06	*TLN1, PTEN, VCL, PAK6, CDC42, PDPK1, ARHGAP5, PAK2, PAK3, PAK4, RHOA, PDGFC, SHC1, SHC2, AKT3, PRKCA, PARVG, ROCK1,* ***BRAF****, PIK3CB, MYLK4, ACTN2, FLNB, CCND1, JUN, VEGFA, PDGFRB, LAMC2, LAMC1, COL1A1, PARVA, CAV2, DIAPH1, TNC, PPP1R12B, ITGB4, SRC, IGF1R, BCL2, COL27A1, ITGAV, SOS1, RAC1, SOS2, PPP1R12A, TNN, PIK3R3, THBS2, FN1, COL4A2, VAV3, FLT1, COL4A1, MET, ITGA1, IGF1, ITGA3, ITGA4, CAPN2, COL5A1, LAMA4, ITGA6, GSK3B, RAP1B, MYLK*
hsa05215	Prostate cancer	5.97e-06	2.94e-04	*TLN1, PTEN, VCL, PAK6, CDC42, PDPK1, ARHGAP5, PAK2, PAK3, PAK4, RHOA, PDGFC, SHC1, SHC2, AKT3, PRKCA, PARVG, ROCK1,* ***BRAF****, PIK3CB, MYLK4, ACTN2, FLNB, CCND1, JUN, VEGFA, PDGFRB, LAMC2, LAMC1, COL1A1, PARVA, CAV2, DIAPH1, TNC, PPP1R12B, ITGB4, SRC, IGF1R, BCL2, COL27A1, ITGAV, SOS1, RAC1, SOS2, PPP1R12A, TNN, PIK3R3, THBS2, FN1, COL4A2, VAV3, FLT1, COL4A1, MET, ITGA1, IGF1, ITGA3, ITGA4, CAPN2, COL5A1, LAMA4, ITGA6, GSK3B, RAP1B, MYLK*
hsa04810	Regulation of actin cytoskeleton	7.45e-06	2.94e-04	*TLN1, PTEN, VCL, PAK6, CDC42, PDPK1, ARHGAP5, PAK2, PAK3, PAK4, RHOA, PDGFC, SHC1, SHC2, AKT3, PRKCA, PARVG, ROCK1,* ***BRAF****, PIK3CB, MYLK4, ACTN2, FLNB, CCND1, JUN, VEGFA, PDGFRB, LAMC2, LAMC1, COL1A1, PARVA, CAV2, DIAPH1, TNC, PPP1R12B, ITGB4, SRC, IGF1R, BCL2, COL27A1, ITGAV, SOS1, RAC1, SOS2, PPP1R12A, TNN, PIK3R3, THBS2, FN1, COL4A2, VAV3, FLT1, COL4A1, MET, ITGA1, IGF1, ITGA3, ITGA4, CAPN2, COL5A1, LAMA4, ITGA6, GSK3B, RAP1B, MYLK*
hsa04010	MAPK signaling pathway	1.42e-05	4.80e-04	*MEF2C, FGF5, FGF18, FGF9, FGF14, FGF10, NFKB1, FGF12, DAXX, TGFB2, MAP3K7, CDC42, PAK2, MAP3K8, PRKACB, RAPGEF2, MAP2K7, AKT3, PRKCA,* ***BRAF****, TP53, CACNG3, FLNB, STK3, RASGRF2, ARRB1, JUN, PDGFRB, STMN1, MAP3K14, FGFR1, MRAS, MKNK2, PPM1A, DUSP10, CACNB2, MAPKAPK2, PPM1B, SRF, ATF2, MAP3K3, ELK4, MAP3K2, SOS1, MAP3K1, RASGRP1, SOS2, RAC1, NFATC3, TAOK1, MAP2K3, TGFBR1, NF1, TGFBR2, TAB2, RPS6KA6, RPS6KA3, RPS6KA4, DUSP1, RPS6KA2, NTRK2, CACNA1E, RAP1B, DUSP8, CACNA1A, CACNA1B, DUSP6*
hsa04722	Neurotrophin signaling pathway	2.59e-5	7.69e-04	*ZNF274, NFKB1, MAPKAPK2, FOXO3, CDC42, PDPK1, MAP3K3, BCL2, SOS1, MAP3K1, RAC1, SOS2, RHOA, SH2B3, SHC1, SH2B1, CALML5, PIK3R3, FRS2, SHC2, MAP2K7, AKT3, ARHGDIA,* ***BRAF****, PIK3CB, TP53, PTPN11, NTRK3, RPS6KA6, RPS6KA3, PSEN1, CAMK4, RPS6KA2, JUN, GSK3B, NTRK2, SORT1, RAP1B*
hsa05218	Melanoma	3.89e-04	0.0057	*FGF5, FGF18, FGFR1, E2F3,* ***BRAF****, FGF9, FGF14, PIK3CB, MET, TP53, IGF1, FGF10, CDH1, CDK6, FGF12, PTEN, IGF1R, CCND1, CDKN2A, MDM2, PDGFRB, PDGFC, PIK3R3, AKT3*
hsa05211	Renal cell carcinoma	3.29e-04	0.0051	***BRAF****, EPAS1, PIK3CB, MET, EGLN3, PTPN11, ARNT, TGFB2, PAK6, CDC42, EP300, PAK2, PAK3, ETS1, JUN, SOS1, PAK4, RAC1, SOS2, VEGFA, RAP1B, PIK3R3, AKT3*
hsa04015	Rap1 signaling pathway	6.53e-05	0.0015	*FGF18, FGF5, ADCY1, TLN1, FGF14, FGF9, EFNA1, ADCY5, ADCY6, FGF10, LPAR1, FGF12, CDC42, PLCB4, RHOA, RALB, PDGFC, AFDN, RAPGEF2, PLCB1, ANGPT2, AKT3, PRKCA, MAGI1,* ***BRAF****, PIK3CB, GRIN2A, SIPA1L3, GNAQ, VEGFA, PDGFRB, EFNA5, GNAS, PRKD3, FGFR1, DRD2, GNAI1, CSF1, MRAS, KITLG, CDH1, SRC, IGF1R, CNR1, P2RY1, RAC1, CALML5, PIK3R3, GNAO1, FLT1, MAP2K3, MET, IGF1, RALGDS, ADCY9, RAP1B*
hsa05205	Proteoglycans in cancer	3.08e-05	8.12e-04	*MMP9, MMP2, IQGAP1, SDC2, TGFB2, CDC42, WNT4, PDPK1, CD44, ANK2, ANK3, RHOA, PRKACB, WNT6, FRS2, AKT3, TWIST1, PRKCA, ROCK1,* ***BRAF****, PIK3CB, TP53, ESR1, ARHGEF12, FLNB, CCND1, SDC1, VEGFA, MDM2, CAV2, FGFR1, PPP1R12B, MRAS, RDX, SRC, IGF1R, ITGAV, SOS1, RAC1, SOS2, PPP1R12A, PIK3R3, WNT8A, FN1, FZD8, MET, CBL, IGF1, IGF2, FZD3, FZD5, FZD6, PTPN11, PTCH1, SLC9A1*
hsa05220	Chronic myeloid leukemia	4.87e-04	0.0067	*E2F3,* ***BRAF****, PIK3CB, TGFBR1, CBL, TGFBR2, TP53, SMAD4, NFKB1, CDK6, PTPN11, TGFB2, CCND1, HDAC2, CDKN2A, GAB2, SOS1, SOS2, MDM2, SHC1, PIK3R3, RUNX1, SHC2, AKT3*
hsa05212	Pancreatic cancer	7.15e-04	0.0089	*E2F3,* ***BRAF****, PIK3CB, ARHGEF6, TGFBR1, TGFBR2, TP53, SMAD4, NFKB1, CDK6, RALGDS, RAD51, TGFB2, CDC42, CCND1, CDKN2A, RAC1, VEGFA, RALB, JAK1, PIK3R3, AKT3*
hsa05210	Colorectal cancer	0.0025	0.0215	*DCC, TCF7,* ***BRAF****, MSH2, PIK3CB, TGFBR1, TGFBR2, TP53, SMAD4, TCF7L1, RALGDS, TGFB2, CCND1, JUN, BCL2, GSK3B, RAC1, RHOA, PIK3R3, AKT3*
hsa05214	Glioma	0.0045	0.0347	*PRKCA, E2F3,* ***BRAF****, PIK3CB, TP53, IGF1, CDK6, PTEN, IGF1R, CCND1, CDKN2A, SOS1, SOS2, MDM2, PDGFRB, SHC1, CALML5, PIK3R3, SHC2, AKT3*
hsa05213	Endometrial cancer	0.0051	0.0369	*TCF7,* ***BRAF****, PIK3CB, TP53, CDH1, FOXO3, CTNNA1, PTEN, CTNNA3, TCF7L1, PDPK1, CCND1, GSK3B, SOS1, SOS2, PIK3R3, AKT3*
hsa05216	Thyroid cancer	0.0107	0.0652	*CCDC6, CCND1, TCF7, RET,* ***BRAF****, RXRA, PAX8, TP53, CDH1, TCF7L1, TPM3*

KEGG ID	Pathway	*P*-value	FDR	All target genes within the pathway (including the genes of interest in *bold*)
hsa04068	FoxO signaling pathway	0.0165	0.0791	*RAG1, FOXO1, FOXO3, PTEN, TGFB2, IGF1R, PDPK1, SLC2A4, SOS1, SOS2, BCL6, PRKAA1, PIK3R3, AKT3, IL6, **BRAF**, SGK3, PIK3CB, TGFBR1, TGFBR2, SMAD4, IGF1, HOMER1, SIRT1, BCL2L11, SOD2, CCND1, EP300, PLK2, FOXG1, SETD7, MDM2*
hsa04024	cAMP signaling pathway	0.0166	0.0791	*PPARA, ATP1B1, ADCY1, GNAI1, DRD2, ADCY5, ADCY6, PDE3B, OXTR, NFKB1, GABBR2, ATP2B2, ATP2B3, ATP2B4, RAC1, PPP1R12A, RHOA, CREB3L2, AFDN, PRKACB, CALML5, HCAR3, PIK3R3, HCAR2, AKT3, HCN2, PLD1, VAV3, **BRAF**, ROCK1, PIK3CB, CREB1, GRIN2A, PDE4D, CREB5, EP300, ADCY9, CAMK4, GRIA1, JUN, PTCH1, RAP1B, GNAS, SLC9A1*
hsa05219	Bladder cancer	0.0216	0.0920	*E2F3, CCND1, CDKN2A, **BRAF**, MMP9, VEGFA, TP53, MDM2, CDH1, MMP2, SRC, MMP1, DAPK1*
hsa04150	mTOR signaling pathway	0.0332	0.1213	*PRKAA1, PDPK1, PTEN, PIK3R3, **BRAF**, PRKCA, TSC1, PIK3CB, IGF1, RPS6KA3, RPS6KA6, RPS6KA2, AKT3, ULK2, RICTOR, EIF4E*
**hsa-miR-708-3p**				
hsa04150	mTOR signaling pathway	0.0101	1.0000	*EIF4E, **BRAF**, TSC1, PRKAA2, MLST8, AKT3, PIK3R1, RPTOR*
hsa04068	FoxO signaling pathway	0.0244	1.0000	*PRKAG3, IGF1R, **BRAF**, SGK3, CCND2, SOS1, SMAD2, PRKAA2, C8ORF44-SGK3, AKT3, PIK3R1, BCL2L11*
hsa04010	MAPK signaling pathway	0.0361	1.0000	***BRAF**, CACNG8, TAOK3, MAP2K4, GNG12, DUSP5, CDC42, PAK2, RASGRP3, SOS1, DUSP16, CACNA1H, RAP1B, FAS, MAP3K14, TRAF6, FGF2, AKT3*
hsa05200	Pathways in cancer	0.0391	1.0000	*GNA13, HSP90AB1, AR, APC2, **BRAF**, FLT3, SMAD2, GNG12, TPM3, CCNE2, EDNRB, CCDC6, IGF1R, CDC42, RASGRP3, LPAR5, SOS1, GNB4, PTCH1, FAS, TRAF6, FGF2, AKT3, PIK3R1, TRAF3*
**hsa-miR-222-3p**				
hsa04012	ErbB signaling pathway	1.64e-07	3.63-05	*NRG4, ERBB4, ERBB3, STAT5A, SRC, PTK2, PAK2, PAK3, GAB1, PAK5, TGFA, CAMK2B, SHC1, PAK1, SHC3, NRG1, MYC, AKT3, PIK3R1, PRKCA, EGFR, MAP2K1, **BRAF**, PIK3CB, MAP2K4, PIK3CD, CBL, MAPK10, MAPK1, CDKN1B, CRKL, GSK3B, PLCG2, MAPK3, ABL2*
hsa04510	Focal adhesion	5.51e-06	5.83e-04	*TLN1, PDGFA, VTN, PTEN, VCL, ACTG1, PAK2, PAK3, PAK5, ILK, RHOA, PDGFC, SHC1, ZYX, PDGFD, PAK1, SHC3, AKT3, PRKCA, EGFR, **BRAF**, PIK3CB, MYLK4, PIK3CD, FLNB, FLNA, VEGFB, MAPK1, CRKL, RASGRF1, CCND2, MAPK3, LAMC2, PARVA, ITGA10, ITGB3, SRC, PTK2, COL6A6, COL6A5, ITGB8, BCL2, THBS1, THBS2, PIK3R1, COL4A4, ACTB, COL4A1, MAP2K1, IGF1, MAPK10, BIRC3, VAV2, COL5A2, COL4A6, KDR, GSK3B, RAP1A, RAP1B*
hsa05205	Proteoglycans in cancer	9.57e-06	5.83e-04	*VTN, HOXD10, SDC2, ACTG1, CTTN, WNT3, ANK1, ANK2, TIAM1, HPSE, ANK3, GAB1, RHOA, PAK1, MSN, MYC, FRS2, AKT3, PRKCA, EGFR, **BRAF**, PIK3CB, PIK3CD, TP53, ESR1, ARHGEF12, FLNB, FLNA, MAPK1, SDC1, MAPK3, WNT9B, MDM2, ERBB4, ERBB3, MRAS, RDX, ITGB3, TIMP3, SRC, PTK2, EZR, HPSE2, CAMK2B, THBS1, PIK3R1, ACTB, FZD9, MAP2K1, CBL, IGF1, ITPR2, KDR, PTPN11, EIF4B, MAPK14, PLCG2*
hsa04015	Rap1 signaling pathway	1.05e-05	5.83e04	*FGF5, ADCY1, TLN1, ADCY2, EFNA1, PDGFA, EFNA3, FGF12, ACTG1, PLCB4, GRIN2B, TIAM1, RHOA, RAPGEF5, RALA, PDGFC, PDGFD, RAPGEF2, INSR, AKT3, ANGPT4, PRKCA, EGFR, MAGI2, MAGI1, **BRAF**, PIK3CB, PIK3CD, SIPA1L2, VEGFB, MAPK1, CRKL, KRIT1, MAPK3, GNAI3, GNAI2, MRAS, CSF1, KITLG, CDH1, ITGB3, KIT, SRC, PFN1, CNR1, THBS1, PIK3R1, ACTB, PARD6B, GNAO1, MAP2K1, IGF1, KDR, RASSF5, MAPK14, RAP1A, RAP1B, PARD6G, CALM1*
hsa05213	Endometrial cancer	7.59e-05	0.0028	*EGFR, MAP2K1, **BRAF**, PIK3CB, PIK3CD, TP53, CDH1, FOXO3, TCF7L2, PTEN, CTNNA3, CTNNA2, MAPK1, CASP9, GSK3B, ILK, MAPK3, AXIN2, MYC, PIK3R1, AKT3*
hsa04722	Neurotrophin signaling pathway	8.02e-05	0.0028	*FOXO3, BCL2, GAB1, RHOA, CAMK2B, SHC1, TRAF6, SHC3, FRS2, AKT3, PIK3R1, IRAK2, IRAK1, NTF3, **BRAF**, MAP2K1, PIK3CB, PIK3CD, TP53, MAPK10, KIDINS220, YWHAE, TP73, PTPN11, MAPK1, CRKL, RPS6KA1, PSEN1, MAPK14, GSK3B, PLCG2, NTRK2, MAPK3, SORT1, RAP1A, RAP1B, CALM1*
hsa05214	Glioma	1.03e-04	0.0028	*PRKCA, E2F1, EGFR, E2F2, MAP2K1, **BRAF**, PIK3CB, PDGFA, PIK3CD, TP53, IGF1, CDK6, PTEN, MAPK1, PLCG2, MAPK3, TGFA, MDM2, CAMK2B, SHC1, SHC3, PIK3R1, AKT3, CALM1*
hsa05200	Pathways in cancer	2.58e-04	0.0052	*E2F1, FGF5, E2F2, ADCY1, PPARD, ADCY2, PDGFA, GNA11, STAT5A, MITF, FOXO1, FGF12, GLI2, CXCL12, PTEN, MMP1, EDNRA, FOS, WNT3, PLCB4, CASP9, CDKN2B, RHOA, TGFA, RALA, MYC, AKT3, EGFR, PRKCA, PTGER2, HSP90AA1, **BRAF**, PIK3CB, PIK3CD, CYCS, TP53, RUNX1T1, CDK6, DAPK2, ARHGEF12, CTNNA3, RAD51, CTNNA2, VEGFB, MAPK1, CRKL, EP300, MAPK3, WNT9B, MDM2, LAMC2, TRAF2, CKS1B, GNAI3, GNAI2, PML, TFG, KITLG, CDH1, KIT, GNG12, ZBTB16, TCF7L2, TPM3, ARNT, PTK2, BCL2, RASGRP1, TRAF6, AXIN2, PIK3R1, TRAF3, COL4A4, FZD9, DVL2, COL4A1, MAP2K1, TGFBR1, CBL, SMAD4, IGF1, BIRC5, MAPK10, BIRC3, COL4A6, RASSF5, CDKN1B, ETS1, GSK3B, PLCG2*

KEGG ID	Pathway	*P*-value	FDR	All target genes within the pathway (including the genes of interest in *bold*)
hsa05210	Colorectal cancer	4.07e-04	0.0061	*MAP2K1, **BRAF**, PIK3CB, TGFBR1, CYCS, PIK3CD, TP53, SMAD4, BIRC5, MAPK10, TCF7L2, MAPK1, FOS, CASP9, GSK3B, BCL2, MAPK3, RHOA, AXIN2, MYC, PIK3R1, AKT3*
hsa05215	Prostate cancer	4.08e-04	0.0061	*E2F1, E2F2, PDGFA, FOXO1, TCF7L2, PTEN, CASP9, BCL2, TGFA, PDGFC, PDGFD, PIK3R1, AKT3, EGFR, HSP90AA1, MAP2K1, **BRAF**, PIK3CB, CREB1, PIK3CD, TP53, IGF1, MAPK1, CDKN1B, EP300, GSK3B, MAPK3, MDM2*
hsa04068	FoxO signaling pathway	4.12e-04	0.0061	*PRKAG3, FOXO1, FOXO3, IL7R, PTEN, LOC400927-CSNK1E, CDKN2B, PRKAA1, INSR, AKT3, PIK3R1, IRS4, EGFR, **BRAF**, MAP2K1, PIK3CB, NLK, TGFBR1, PRKAB2, PIK3CD, SMAD4, IGF1, MAPK10, GRM1, SIRT1, BCL2L11, SOD2, MAPK1, TNFSF10, EP300, CDKN1B, CSNK1E, PLK1, CCND2, MAPK14, MAPK3, MDM2, GADD45B*
hsa05223	Non-small cell lung cancer	7.40e-04	0.0082	*PRKCA, E2F1, EGFR, FHIT, E2F2, **BRAF**, MAP2K1, PIK3CB, PIK3CD, TP53, CDK6, FOXO3, MAPK1, RASSF5, CASP9, PLCG2, MAPK3, TGFA, PIK3R1, AKT3*
hsa05218	Melanoma	0.0011	0.0100	*E2F1, EGFR, FGF5, E2F2, MAP2K1, **BRAF**, PIK3CB, PDGFA, MITF, PIK3CD, TP53, IGF1, CDH1, CDK6, FGF12, PTEN, MAPK1, MAPK3, MDM2, PDGFC, PDGFD, PIK3R1, AKT3*
hsa05220	Chronic myeloid leukemia	0.0014	0.0114	*E2F1, E2F2, MAP2K1, **BRAF**, PIK3CB, TGFBR1, STAT5A, CBL, PIK3CD, TP53, SMAD4, CDK6, PTPN11, MAPK1, CRKL, CDKN1B, MAPK3, MDM2, SHC1, SHC3, MYC, AKT3, PIK3R1*
hsa05211	Renal cell carcinoma	0.0026	0.0181	*MAP2K1, **BRAF**, PIK3CB, PIK3CD, PTPN11, ARNT, MAPK1, EP300, CRKL, PAK2, PAK3, ETS1, GAB1, PAK5, MAPK3, TGFA, RAP1A, RAP1B, PAK1, PIK3R1, AKT3*
hsa04024	cAMP signaling pathway	0.0027	0.0184	*PPARA, ATP1B1, ADCY1, GNAI3, ADCY2, GNAI2, ADCYAP1R1, FFAR2, ATP1B4, GABBR1, GABBR2, CNGB1, VIPR2, EDNRA, FOS, ATP2B3, ATP2B4, GRIN2B, TIAM1, PDE4B, RHOA, CAMK2B, PAK1, HCN4, AKT3, PIK3R1, PTGER2, **BRAF**, MAP2K1, PIK3CB, CREB1, PIK3CD, PDE3A, PDE4D, ATP1A1, GRIA4, MAPK10, VAV2, CACNA1S, MAPK1, EP300, GRIA1, CHRM1, MAPK3, RAP1A, RYR2, RAP1B, CALM1*
hsa05219	Bladder cancer	0.0033	0.0219	*EGFR, E2F1, E2F2, **BRAF**, MAP2K1, TP53, CDH1, DAPK2, SRC, MMP1, MAPK1, MAPK3, MDM2, THBS1, MYC*
hsa04010	MAPK signaling pathway	0.0036	0.0230	*FGF5, PDGFA, FGF12, DAXX, FOS, PAK2, PAK1, RAPGEF2, MYC, AKT3, PRKCA, EGFR, **BRAF**, TP53, FLNB, FLNA, MAPK1, CRKL, RASGRF1, MAPK3, STMN1, GADD45B, TRAF2, IL1R1, MRAS, PPP3R1, PPM1A, CACNB2, CACNB3, GNG12, CACNB4, PPM1B, ATF2, ELK4, MAP3K2, RASGRP1, DUSP16, PPP3CB, TRAF6, NFATC3, HSPA8, MAP2K1, NTF3, TAOK1, NLK, TGFBR1, MAP2K4, MAPK10, CACNA1S, DUSP4, DUSP3, RPS6KA1, MAPK14, NTRK2, MAPK8IP2, RAP1A, RAP1B, PLA2G4C*
hsa05212	Pancreatic cancer	0.0051	0.0299	*E2F1, EGFR, E2F2, **BRAF**, MAP2K1, PIK3CB, TGFBR1, PIK3CD, TP53, SMAD4, CDK6, MAPK10, RAD51, MAPK1, CASP9, MAPK3, TGFA, RALA, PIK3R1, AKT3*
hsa04910	Insulin signaling pathway	0.0054	0.0302	*PRKAG3, EXOC7, FOXO1, RHOQ, PRKAR2A, FASN, PRKAA1, SHC1, SHC3, INSR, AKT3, PIK3R1, IRS4, **BRAF**, MAP2K1, SOCS3, PIK3CB, PHKG2, PRKAB2, PIK3CD, CBL, SOCS1, ACACA, SOCS4, MAPK10, PPARGC1A, MAPK1, CRKL, EIF4E, TSC1, GSK3B, MAPK3, EIF4E2, PYGB, CALM1*
hsa04150	mTOR signaling pathway	0.0076	0.0364	*PRKCA, **BRAF**, PIK3CB, PIK3CD, IGF1, RRAGD, PTEN, DDIT4, EIF4B, MAPK1, EIF4E, RPS6KA1, TSC1, MAPK3, PRKAA1, EIF4E2, PIK3R1, AKT3*
hsa04810	Regulation of actin cytoskeleton	0.0218	0.0769	*CHRM1, SRC, ITGB3, PDGFA, BRK1, PIK3CD, PIK3CB, PIK3R1, EGFR, ACTB, ACTG1, CRKL, MYLK4, FGF5, PAK1, PDGFD, PDGFC, PIP5K1A, MAPK1, ITGB8, PAK3, PAK2, WASF2, PAK5, MAPK3, MAP2K1, ARHGEF12, RDX, MSN, **BRAF**, ARPC4, SSH2, GNG12, RHOA, PTK2, VAV2, ENAH, TIAM1, MRAS, ABI2, ITGA10, EZR, PFN1, ARHGEF7, FGF12, VCL*
hsa05221	Acute myeloid leukemia	0.0269	0.0881	*STAT5A, TCF7L2, MAP2K1, ZBTB16, PIK3CD, **BRAF**, PIK3CB, PIK3R1, PML, MYC, KIT, AKT3, MAPK1, PPARD, MAPK3, RUNX1T1*
hsa05216	Thyroid cancer	0.0326	0.1020	*TCF7L2, MAP2K1, TFG, TPM3, CDH1, MYC, MAPK1, **BRAF**, TP53, MAPK3*
hsa04062	Chemokine signaling pathway	0.0423	0.1269	*CX3CR1, GSK3B, CXCL9, CCL11, SHC3, SHC1, SRC, GNAI3, PIK3CD, ADCY2, ADCY1, PIK3CB, PIK3R1, FOXO3, CXCL14, GNAI2, CRKL, RAP1B, PAK1, RAP1A, CCL5, AKT3, GRK6, MAPK1, JAK3, MAPK3, CCR1, LYN, MAP2K1, CCL22, STAT2, **BRAF**, GNG12, RHOA, PTK2, VAV2, TIAM1, PLCB4, CXCL12, CCL28*
				**hsa-miR-146b-3p**
hsa04015	Rap1 signaling pathway	1.38e-04	0.0109	*ADCY1, ADCY2, PDGFB, PGF, FGF14, BCAR1, FGF17, ADCY5, ADCY6, FGF12, CTNNB1, ACTG1, GRIN2B, PDGFD, RAPGEF3, ANGPT2, AKT2, PRKCA, **BRAF**, PRKCI, SIPA1L3, PRKCB, MAPK1, CRKL, NGFR, GNAI3, GNAI2, ADORA2A, DRD2, MRAS, CSF1, ITGB3, PFN2, KRAS, RASGRP3, PIK3R3, PIK3R1, GNAO1, MAP2K1, MAP2K3, MET, RAF1, HGF, RALGDS, RASSF5, PLCG1, ADCY9, CALM3, CALM1*

KEGG ID	Pathway	*P*-value	FDR	All target genes within the pathway (including the genes of interest in *bold*)
hsa05223	Non-small cell lung cancer	2.71e-04	0.0133	*PRKCA, FHIT, E2F2,* ***BRAF****, MAP2K1, TP53, RAF1, CDK6, PRKCB, MAPK1, RASSF5, CCND1, KRAS, PLCG1, CASP9, PLCG2, PIK3R3, PIK3R1, AKT2*
hsa05210	Colorectal cancer	3.69e-04	**0.0133**	***BRAF****, MAP2K1, MSH2, TP53, TGFB3, RAF1, SMAD3, BIRC5, SMAD2, RALGDS, CTNNB1, MAPK1, CCND1, KRAS, CASP9, GSK3B, MAPK9, PIK3R3, PIK3R1, AKT2*
hsa04012	ErbB signaling pathway	3.92e-04	**0.0133**	*PRKCA, ERBB4,* ***BRAF****, MAP2K1, MAP2K4, CBL, RAF1, PRKCB, MAPK1, KRAS, CRKL, CDKN1B, PLCG1, EREG, PAK3, GSK3B, PLCG2, MAPK9, PIK3R3, SHC3, NRG1, NRG2, ABL2, PIK3R1, AKT2*
hsa05214	Glioma	7.12e-04	0.0188	*PRKCA, E2F2, PDGFB,* ***BRAF****, MAP2K1, TP53, RAF1, CDK6, PRKCB, MAPK1, CCND1, KRAS, PLCG1, PLCG2, CALM3, PIK3R3, SHC3, PIK3R1, AKT2, CALM1*
hsa05212	Pancreatic cancer	7.12e-04	0.0188	*E2F2,* ***BRAF****, MAP2K1, RELA, TP53, TGFB3, RAF1, SMAD3, BRCA2, SMAD2, CDK6, RALGDS, MAPK1, CCND1, KRAS, CASP9, MAPK9, PIK3R3, PIK3R1, AKT2*
hsa05200	Pathways in cancer	0.0010	0.0210	*GNA13, E2F2, ADCY1, PPARD, ADCY2, PDGFB, PGF, FGF14, ADCY5, FGF17, ADCY6, ARNT2, TGFB3, FGF12, MMP2, CXCL12, CTNNB1, CCNE1, CASP9, PRKACA, NOS2, WNT6, AKT2, PRKCA, AR, PTGER3, HSP90AA1,* ***BRAF****, RELA, TP53, CDK6, CTNNA1, ARHGEF12, PRKCB, MAPK1, CCND1, CRKL, MAPK9, WNT9A, TRAF1, GNAI3, GNAI2, PML, CXCL8, GNG12, ZBTB16, BDKRB2, SUFU, TPM3, ARNT, KRAS, RASGRP3, PIK3R3, PIK3R1, TRAF3, MAP2K1, MSH2, MET, CBL, RAF1, SMAD3, BRCA2, BIRC5, SMAD2, FZD2, HGF, RALGDS, GNGT1, RASSF5, WNT7B, FZD10, CDKN1B, ADCY9, PLCG1, GSK3B, PLCG2*
hsa05215	Prostate cancer	0.0027	0.0353	*E2F2, AR, HSP90AA1, MAP2K1,* ***BRAF****, PDGFB, RELA, TP53, RAF1, CREB5, CTNNB1, CCNE1, MAPK1, CCND1, KRAS, CDKN1B, CASP9, GSK3B, CREB3L2, PDGFD, PIK3R3, PIK3R1, AKT2*
hsa04722	Neurotrophin signaling pathway	0.0048	0.0457	*IRAK3, KRAS, SH2B1, SHC3, PIK3R3, PIK3R1, ARHGDIB, AKT2, IRAK1, MAP2K1,* ***BRAF****, RELA, TP53, RAF1, KIDINS220, NTRK3, RPS6KA6, MAPK1, CRKL, PLCG1, GSK3B, PLCG2, PSEN2, MAPK9, CALM3, SORT1, NGFR, CALM1*
hsa05218	Melanoma	0.0054	0.0481	*E2F2, PDGFB,* ***BRAF****, MAP2K1, FGF14, FGF17, MET, TP53, RAF1, CDK6, HGF, FGF12, MAPK1, CCND1, KRAS, PDGFD, PIK3R3, PIK3R1, AKT2*
hsa05220	Chronic myeloid leukemia	0.0063	0.0506	*E2F2,* ***BRAF****, MAP2K1, RELA, CBL, TP53, TGFB3, RAF1, CDK6, MAPK1, CCND1, CRKL, KRAS, CDKN1B, GAB2, PIK3R3, SHC3, PIK3R1, AKT2*
hsa04150	mTOR signaling pathway	0.0088	0.0594	*PRKCA, CAB39L,* ***BRAF****, RICTOR, RPTOR, EIF4E1B, PRKCB, RPS6KA6, MAPK1, TSC1, PRKAA1, CAB39, MLST8, PIK3R3, PIK3R1, AKT2*
hsa04024	cAMP signaling pathway	0.0093	0.0599	*PPARA, ADCY1, GNAI3, ADCY2, GNAI2, ADORA2A, DRD2, ADCY5, ADCY6, GRIN3A, VIPR2, GRIN2B, CREB3L2, PRKACA, RAPGEF3, HCAR3, PIK3R3, HCAR2, PIK3R1, AKT2, PLD1, PTGER3, MAP2K1,* ***BRAF****, RELA, RAF1, PDE4D, CREB5, NPY1R, SSTR5, MAPK1, ADCY9, GRIA1, GIPR, MAPK9, ABCC4, CALM3, GHSR, GLP1R, CALM1*
hsa04062	Chemokine signaling pathway	0.0158	0.0899	*ADCY1, GNAI3, ADCY2, GNAI2, FGR, CCR1, BCAR1, ADCY5, ADCY6, CXCL8, CXCR2, GNG12, CXCL12, CCL28, CCL24, CCL22, KRAS, CXCR5, PRKACA, SHC3, PIK3R3, PIK3R1, AKT2,* ***BRAF****, MAP2K1, RELA, RAF1, CCL4L2, CCL17, CCR9, MAPK1, GNGT1, CCR7, CRKL, ADCY9, CCR4, GSK3B*
hsa05213	Endometrial cancer	0.0189	0.1000	***BRAF****, MAP2K1, TP53, RAF1, CTNNA1, CTNNB1, MAPK1, CCND1, KRAS, CASP9, GSK3B, PIK3R3, PIK3R1, AKT2*
hsa04810	Regulation of actin cytoskeleton	0.0234	0.1188	*GNA13, ENAH, PDGFB, FGF14, MRAS, PPP1R12B, BCAR1, FGF17, WASF2, PIP5K1C, ABI2, RDX, GNG12, FGF12, PIP5K1A, ITGB3, BDKRB2, VCL, ACTG1, PFN2, KRAS, PAK3, ITGB6, PIKFYVE, PDGFD, PIK3R3, PIK3R1,* ***BRAF****, MAP2K1, LIMK1, ARHGEF7, BAIAP2, MYLK4, RAF1, ARHGEF12, MAPK1, CRKL, SCIN, CYFIP2, PIP4K2C*
hsa05205	Proteoglycans in cancer	0.0282	0.1323	*CAV3, ERBB4, MRAS, PPP1R12B, RDX, ITGB3, MMP2, PDCD4, CTNNB1, ACTG1, CTTN, KRAS, CD44, PRKACA, WNT6, PIK3R3, PIK3R1, AKT2, PRKCA, MAP2K1,* ***BRAF****, CBL, MET, TP53, RAF1, FZD2, HGF, ARHGEF12, PRKCB, ITPR2, MAPK1, CCND1, SDC1, WNT7B, FZD10, PLCG1, PLCG2, WNT9A*
hsa05211	Renal cell carcinoma	0.0283	0.1323	*PDGFB,* ***BRAF****, MAP2K1, ARNT2, MET, TGFB3, RAF1, HGF, ARNT, MAPK1, CRKL, KRAS, PAK3, PIK3R3, PIK3R1, AKT2*
hsa04910	Insulin signaling pathway	0.0296	0.1329	*G6PC2, PPP1R3E, PRKAR2A, KRAS, GYS1, PRKACA, PRKAA1, SHC3, PIK3R3, TRIP10, PIK3R1, AKT2, MAP2K1,* ***BRAF****, CBL, PRKAB1, PRKCI, RAF1, RPTOR, EIF4E1B, MAPK1, G6PC, CRKL, TSC1, GSK3B, MAPK9, CALM3, CALM1*
hsa05221	Acute myeloid leukemia	0.0338	0.1411	*MAPK1, CCND1, PPARD, KRAS, MAP2K1,* ***BRAF****, RELA, PML, RAF1, PIM2, ZBTB16, PIK3R3, PIK3R1, AKT2*
				**hsa-miR-31-5p**

KEGG ID	Pathway	*P*-value	FDR	All target genes within the pathway (including the genes of interest in *bold*)
hsa05200	Pathways in cancer	3.04e-05	0.0080	*ADCY3, GNA13, FGF19, F2RL3, PDGFB, STAT5B, ADCY6, CXCL12, CTNNB1, WNT1, RALB, RALA, GNG3, GNG7, EGFR, RET, **BRAF**, ROCK2, RXRB, FGF23, FADD, STK4, CTNNA3, CTNNA2, CCDC6, JUN, VEGFA, PDGFRB, MAPK8, FGFR3, GNAI3, KRAS, ITGAV, RAC1, AXIN1, TGFBR2, MET, SMAD4, ITGA2, SMAD2, HDAC2, ETS1, PLCG2, ABL1, FGF5, E2F2, FGF7, FGF9, MITF, MLH1, GLI2, GLI3, PTEN, EDNRA, CCNE2, EDNRB, CDC42, CASP3, CASP9, RHOA, HHIP, PLCB1, MYC, AR, PTGER3, RUNX1T1, CDK6, ARHGEF12, CDK2, PRKCB, GNAQ, NCOA4, WNT9B, DCC, EGLN3, PML, KITLG, EGLN1, CDH1, KIT, ZBTB16, TCF7L1, SUFU, TPM3, IGF1R, SOS1, PIK3R1, CEBPA, COL4A2, CBL, FZD1, FZD3, BAD, MAPK10, BIRC3, STAT1, FZD4, HSP90B1, GSK3B, JAK1, CRK*
hsa04510	Focal adhesion	5.51e-04	0.0209	*PDGFB, TLN2, PTEN, CTNNB1, PAK6, CDC42, PDPK1, ARHGAP5, ILK, RHOA, PDGFC, PAK1, SHC4, EGFR, **BRAF**, ROCK2, MYLK4, PPP1CC, PPP1CB, FLNA, PRKCB, CCND2, JUN, VEGFA, PDGFRB, MAPK8, PARVA, DIAPH1, PPP1R12B, PPP1R12C, ITGA11, SRC, IGF1R, DOCK1, ITGB8, ITGAV, SOS1, RAC1, THBS2, PIK3R1, COL4A2, FLT1, VAV3, MET, ITGA2, BAD, MAPK10, BIRC3, COL5A1, ITGA5, ITGA8, GSK3B, RAP1A, RAP1B, CRK, MYLK*
hsa05213	Endometrial cancer	8.07e-04	0.0238	*EGFR, **BRAF**, MLH1, CDH1, BAD, FOXO3, PTEN, CTNNA3, TCF7L1, CTNNB1, CTNNA2, PDPK1, KRAS, CASP9, GSK3B, SOS1, ILK, MYC, PIK3R1, AXIN1*
hsa04810	Regulation of actin cytoskeleton	9.09e-04	0.0241	*GNA13, FGF19, FGF5, FGF7, PDGFB, FGF9, WASF1, WASF2, PIP5K1A, PAK6, CDC42, TIAM1, RHOA, PDGFC, PAK1, EGFR, LIMK2, **BRAF**, ROCK2, ARHGEF7, MYLK4, FGF23, PPP1CC, ARHGEF12, PPP1CB, PDGFRB, FGFR3, SSH1, DIAPH1, DIAPH2, PPP1R12B, PPP1R12C, SSH2, ITGA11, RDX, ARPC5, SRC, PFN2, DOCK1, KRAS, ITGAX, ITGB8, ITGAV, SOS1, RAC1, PIKFYVE, PIK3R1, VAV3, ITGA2, ITGA5, ITGA8, CYFIP1, PIP4K2A, CRK, MYLK, PIP4K2B*
hsa04012	ErbB signaling pathway	0.0013	0.0263	*CAMK2G, STAT5B, SRC, PAK6, KRAS, SOS1, CAMK2D, PAK1, MYC, PIK3R1, SHC4, EGFR, **BRAF**, CBL, MAP2K4, MAPK10, BAD, PRKCB, NCK2, EREG, JUN, GSK3B, PLCG2, HBEGF, MAPK8, ABL1, ABL2, CRK*
hsa05205	Proteoglycans in cancer	0.0025	0.0452	*TLR4, SDC4, SDC2, CTNNB1, CDC42, WNT1, CASP3, PDPK1, TIAM1, RHOA, NUDT16L1, PAK1, FRS2, MYC, EGFR, **BRAF**, ROCK2, PPP1CC, ARHGEF12, PPP1CB, FLNA, PRKCB, SDC1, VEGFA, WNT9B, PPP1R12B, CAMK2G, PPP1R12C, RDX, TIMP3, SRC, IGF1R, KRAS, ITGAV, SOS1, RAC1, CAMK2D, PIK3R1, MET, CBL, FZD1, ITGA2, FZD3, DDX5, FZD4, PTPN11, EIF4B, ITGA5, MAPK14, PLCG2, HBEGF, PLAU*
hsa05210	Colorectal cancer	0.0033	0.0561	*DCC, **BRAF**, TGFBR2, SMAD4, MLH1, SMAD2, BAD, MAPK10, TCF7L1, CTNNB1, CASP3, KRAS, CASP9, JUN, GSK3B, RAC1, RHOA, MAPK8, MYC, PIK3R1, AXIN1*
hsa04722	Neurotrophin signaling pathway	0.0040	0.0561	*CAMK2G, MAPKAPK2, FOXO3, CDC42, PDPK1, KRAS, MAP3K3, MAP3K1, SOS1, RAC1, CAMK2D, RHOA, SH2B3, FRS2, PIK3R1, SHC4, IRAK1, NTF3, **BRAF**, MAPK10, BAD, YWHAE, PTPN11, NTRK3, JUN, MAPK14, GSK3B, PLCG2, RAP1A, MAPK8, RAP1B, ABL1, CRK, CALM1*
hsa04010	MAPK signaling pathway	0.0042	0.0561	*FGF19, FGF5, FGF7, PDGFB, FGF9, MAP3K7, CDC42, CASP3, MAP3K4, MAP3K8, PAK1, MYC, MAP2K6, EGFR, **BRAF**, CACNG4, FGF23, CACNG3, STK4, FLNA, PRKCB, MAP4K4, JUN, PDGFRB, MAPK8, STMN1, MAP3K14, MAP3K13, IL1R1, FGFR3, CACNB1, PPP3R2, CACNB2, CACNB3, PPM1B, MAPKAPK2, SRF, KRAS, MAP3K3, MAP3K1, SOS1, RAC1, PPP3CA, NFATC3, RASA1, CACNA2D1, NTF3, TAOK1, MAP2K4, TGFBR2, NF1, MAPK10, DUSP3, MAPK14, MAPK8IP2, RAP1A, CACNA1E, RAP1B, CRK, DUSP7, CACNA1B, DUSP6*
hsa04015	Rap1 signaling pathway	0.0044	0.0568	*FGF19, ADCY3, FGF5, F2RL3, FGF7, PDGFB, TLN2, FGF9, ADCY6, CTNNB1, CDC42, GRIN2B, TIAM1, RHOA, RALB, RAPGEF5, RALA, PDGFC, PLCB1, MAP2K6, ANGPT4, EGFR, MAGI2, **BRAF**, FGF23, PRKCB, GNAQ, VEGFA, PDGFRB, LCP2, GNAI3, FGFR3, CSF1, FPR1, KITLG, CDH1, KIT, APBB1IP, SRC, IGF1R, PFN2, KRAS, CNR1, RAC1, TEK, PIK3R1, FLT1, MET, MAPK14, RAP1A, RAP1B, CRK, CALM1*
hsa05212	Pancreatic cancer	0.0133	0.1370	*EGFR, E2F2, **BRAF**, TGFBR2, SMAD4, SMAD2, CDK6, BAD, MAPK10, STAT1, CDC42, KRAS, CASP9, VEGFA, RAC1, RALB, RALA, JAK1, MAPK8, PIK3R1*
hsa05216	Thyroid cancer	0.0211	0.2068	*CCDC6, RET, KRAS, **BRAF**, RXRB, NCOA4, CDH1, MYC, TCF7L1, CTNNB1, TPM3*
hsa05211	Renal cell carcinoma	0.0315	0.2641	*PDGFB, **BRAF**, MET, EGLN3, EGLN1, PTPN11, PAK6, CDC42, KRAS, ETS1, JUN, SOS1, RAC1, VEGFA, RAP1A, RAP1B, PAK1, CRK, PIK3R1*
hsa05218	Melanoma	0.0333	0.2687	*FGF19, EGFR, FGF5, E2F2, FGF7, PDGFB, **BRAF**, FGF9, MITF, MET, FGF23, CDH1, CDK6, BAD, PTEN, IGF1R, KRAS, PDGFRB, PDGFC, PIK3R1*

FDR: False Discovery Rate. The hsa-miR-874-3p miRNA is absence since no significant enriched functional pathway involving the *TERT* gene target was found.
